# The inhibitor protein IF_1_ from mammalian mitochondria inhibits ATP hydrolysis but not ATP synthesis by the ATP synthase complex

**DOI:** 10.1016/j.jbc.2024.105690

**Published:** 2024-01-26

**Authors:** Joe Carroll, Ian N. Watt, Charlotte J. Wright, Shujing Ding, Ian M. Fearnley, John E. Walker

**Affiliations:** The Medical Research Council Mitochondrial Biology Unit, University of Cambridge, Cambridge, United Kingdom

**Keywords:** mitochondria, ATP synthase, regulation, inhibitor protein IF_1_, unidirectional inhibition

## Abstract

The hydrolytic activity of the ATP synthase in bovine mitochondria is inhibited by a protein called IF_1_, but bovine IF_1_ has no effect on the synthetic activity of the bovine enzyme in mitochondrial vesicles in the presence of a proton motive force. In contrast, it has been suggested based on indirect observations that human IF_I_ inhibits both the hydrolytic and synthetic activities of the human ATP synthase and that the activity of human IF_1_ is regulated by the phosphorylation of Ser-14 of mature IF_1_. Here, we have made both human and bovine IF_1_ which are 81 and 84 amino acids long, respectively, and identical in 71.4% of their amino acids and have investigated their inhibitory effects on the hydrolytic and synthetic activities of ATP synthase in bovine submitochondrial particles. Over a wide range of conditions, including physiological conditions, both human and bovine IF_1_ are potent inhibitors of ATP hydrolysis, with no effect on ATP synthesis. Also, substitution of Ser-14 with phosphomimetic aspartic and glutamic acids had no effect on inhibitory properties, and Ser-14 is not conserved throughout mammals. Therefore, it is unlikely that the inhibitory activity of mammalian IF_1_ is regulated by phosphorylation of this residue.

In 1963, Pullman and Monroy isolated from bovine mitochondria a low molecular weight protein, now known as IF_1_, or inhibitor of F_1_-ATPase, that, as its name implies, inhibits ATP hydrolysis by the F_1_-catalytic domain of the ATP synthase (1). They demonstrated that in the presence of either of the electron donors NADH or succinate, bovine submitochondrial particles (SMPs), which are everted vesicles of the inner mitochondrial membrane with the F_1_-catalytic domain of the membrane bound ATP synthase extending into the surrounding milieu, generate a proton motive force (pmf) across the vesicular membrane, and that these vesicles were able to make ATP from ADP and phosphate. However, the addition of bovine IF_1_ at a single concentration did not uncouple oxidative phosphorylation (1) and therefore it was concluded that bovine IF_1_ is a unidirectional inhibitor of ATP hydrolysis by the ATP synthase, without effect on the ability of the enzyme to carry out ATP synthesis. Direct single observations of this unidirectional inhibitory activity were made subsequently with purified bovine ATP synthase coreconstituted into phospholipid vesicles with bacteriorhodopsin (2). On provision of light, together with ADP, Mg^2+^, and phosphate, the bacteriorhodopsin generated a transmembrane pmf to drive the synthesis of ATP by the ATP synthase, but, addition of IF_1_ supplied at a single concentration, did not inhibit ATP synthesis. Also, when a pmf is generated in mitochondrial membrane vesicles, IF_1_ disengages from the ATP synthase complex (3), and disengagement from F_1_ occurs effectively only in the presence of ADP and phosphate with the enzyme’s rotor turning in the synthetic direction (4).

Bovine IF_1_ is a basic protein of 84 amino acids (5), and the active form is dimeric with the monomers associated *via* an antiparallel α-helical coiled-coil in their C-terminal regions, and the N-terminal inhibitory regions extending in opposite directions (Fig. 1*A*) (6, 7). In the free solution, these inhibitory regions are intrinsically disordered (8, 9), but in crystals of bovine IF_1_, they are folded into an α-helix (10). In the process of inhibiting the hydrolytic activity of the ATP synthase, each intrinsically disordered inhibitory region interacts initially with the most open of the three catalytic interfaces of the enzyme, the "empty" interface. Then, concomitant with the consecutive hydrolysis of two ATP molecules in each F_1_-domain, the catalytic interfaces close and the inhibitory regions become progressively structured and increasingly enveloped by the enzyme, leading to the final fully inhibited state where each of the inhibitory regions is bound in the "DP" (or diphosphate) interface (10). In this fully inhibited state, each of the inhibitory regions from residues 1 to 13 remains disordered and occupies the central cavity surrounding the α-helical coiled-coil region of the γ-subunit in the central stalk of the enzyme's rotor. Residues 14 to 18 form a short α-helix and together with an extended region from residues 19 to 20 they interact with the α-helical coiled-coil region of the γ-subunit. The extended region is followed by an α-helix from residues 21 to 81. This α-helix includes the bound inhibitory region from residues 21 to 46, which extends unbroken *via* residues 47 to 81 from the surface of the F_1_-catalytic domain. Residues 47 to 81 provide the C-terminal dimerization region (7).

**Figure 1:**
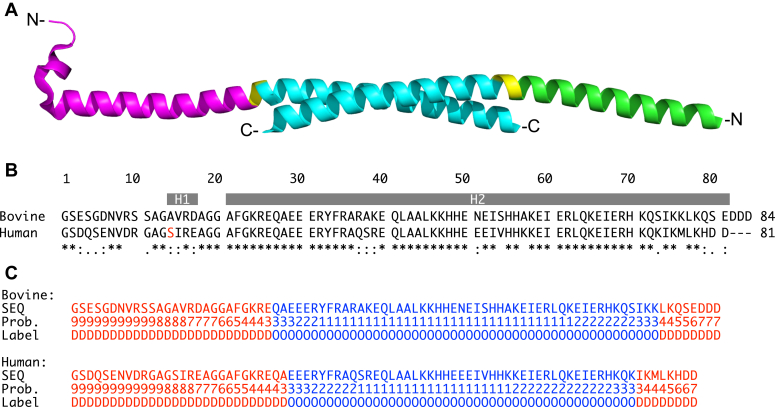
**Structure of bovine IF**_**1**_**and sequence conservation in the bovine and human proteins.***A*, on the *left* is shown, the structure of the monomer of bovine IF_1_ made of 84 amino acids derived from the inhibitory region (residues 11–46 *magenta*) in the crystal structure of monomeric bovine IF_1_ bound to bovine F_1_-ATPase (PDB 4TT3) and the dimerization region (*blue*) from the structure in crystals of isolated dimeric bovine IF_1_ (PDB 1GMJ). The dimerization regions of the two monomers are associated *via* an antiparallel α-helical coiled-coil (residues 49–81). The *right-hand monomer* is taken from the same crystal structure of the isolated dimer, with the inhibitory region colored *green*. The *left-hand monomer* was resolved from residues 11 to 82, and the *right-hand monomer* from residues 20 to 79. The *yellow regions* in each monomer are residues Lys-47 and His-48, found between the inhibitory region and the interacting domain of the coiled-coil. In free solution, the two inhibitory regions are intrinsically disordered (9), and become ordered, as shown in the *left-hand monomer*, on binding to the F_1_-catalytic domain of ATP synthase (10). *B*, comparison of the sequences of bovine and human IF_1_. Sequence conservation is shown above where *asterisks*, *colons*, and *periods*, respectively, indicate identical, strongly conserved, and weakly similar residues. Human residue Ser-14 (*red*) has been proposed to be reversibly phosphorylated (13). The *gray boxes* H1 (residues 14–17) and H2 (residues 21–81) indicate the positions of α-helical regions in the crystal structure of bovine IF_1_ (7). *C*, intrinsically disordered regions (D, *red*) of bovine and human IF_1_ predicted with SPOT-Disorder 2 (57) with the probability scores (Prob.). Regions predicted to be ordered (O) are *blue*.

Until now, the biochemical properties of the closely related human IF_1_ have not been studied *in vitro*, and the assumption that human IF_1_ has a unidirectional inhibitory effect on the human ATP synthase, inhibiting only ATP hydrolysis and not synthesis, has been challenged (11, 12). Thus, it has been asserted that in mitochondria within human cells, IF_1_ inhibits both ATP hydrolysis and synthesis and moreover that reversible phosphorylation of residue Ser-14 of mature IF_1_, referred to previously as residue Ser-39 of the mitochondrial precursor protein, regulates both hydrolytic and synthetic inhibitory activities by preventing IF_1_ from binding to the ATP synthase during either ATP hydrolysis or ATP synthesis (13). The evidence for the inhibition of mitochondrial ATP synthesis by IF_1_ has been questioned previously (14). Here, we have studied the effects of both bovine and human IF_1_ on ATP hydrolysis and ATP synthesis by the ATP synthase in bovine SMPs. For this purpose, we have adopted an established method for assaying ATP (15) and have used this technique to follow the generation of ATP and to explore the impact on this process of various inhibitors, including bovine and human IF_1_ and mutant forms added at a range of molar excesses. The mutant forms of human IF_1_ include those where Ser-14 has been substituted by the phosphomimetic residues, aspartic acid and glutamic acid.

## Results

### Comparison of features of human and bovine IF_1_

The sequences of bovine and human IF_1_ are identical in 71.4% of their amino acids and conservatively substituted in a further 13.1% (Fig. 1*B*). In the inhibitory region, 71.1% of amino acids are identical and a further 17.8% are highly conserved and in the coiled-coil region observed in the bovine dimeric protein (residues 44–84), 73.2% of amino acids are identical in the human ortholog and an additional 7.3% are substituted conservatively. Moreover, the residues of bovine IF_1_ that are involved in binding it to the bovine enzyme are identical, except for residues 11, 15, and 17 where bovine serine, valine, and aspartic acid residues, respectively, are conservatively substituted by human glycine, isoleucine, and glutamic acid. The amino acid residues that bind bovine IF_1_ to the F_1_-domain have been identified by systematic mutation and kinetic analysis (16). They interact predominantly with specific residues in the C-terminal domain of the β-subunit, with another residue in the C-terminal domain of the adjacent α-subunit and with three others in the α-helical coiled-coil region of the γ-subunit (see Table S1). These interacting residues are identical in the human subunits (Table S1), and the sequences of the same three α-, β-, and γ-subunits are identical in 98.4%, 98.6%, and 92.3% of their amino acid residues, respectively (Fig. S1). Another feature of the bovine inhibitor proteins is that its N-terminal region is intrinsically disordered (10), and the same region is predicted to be so in the human protein (Fig. 1*C*). Therefore, it is reasonable to conclude that bovine and human IF_1_ molecules have closely similar structures and properties and that they bind to their cognate F_1_-domains in an essentially identical fashion. Thus, the inhibitory properties of human IF_1_ studied, as they are here, with the more readily available and sufficiently abundant ATP synthase in bovine SMPs, illuminate the binding properties of human IF_1_ with the human ATP synthase.

### ATP synthase content of bovine SMPs

In order to ensure that the investigations of the inhibitory properties included experimental conditions similar to physiological circumstances, the content of ATP synthase in bovine SMPs was determined by absolute quantitative mass spectrometry (MS) using stable isotopically labeled peptide standards. It was found to be 0.48 nmol/mg *n*-dodecyl-β-D-maltoside extract (Table S2), with corresponding molar ratios for the α-, e- and OSCP-subunits of 3.0:0.90:1.2, close to the values 3:1:1 expected from the structure of the enzyme complex (17). The range of molar excesses of IF_1_ employed in the experiments below includes the range of estimated physiological levels, but also extends beyond it (see Table S3).

### Design of inhibitor proteins

All four versions of the bovine inhibitor proteins that were studied contained the mutation Y33W to aid spectroscopic quantitation of the protein. This conservative substitution has no deleterious effect on the inhibitory activity of IF_1_ (16). These four versions of bovine IF_1_ were as follows: the full-length dimeric protein BovIF_1_(1–84)-Y33W, where the ATP hydrolytic inhibitory activity of dimers is regulated by both pH and concentration of cations; BovIF_1_(1–84)-Y33W-H49K, where the mutation H49K makes the protein constitutively active in the inhibition of ATP hydrolysis from pH values below 8 (18); the monomeric inhibitors BovIF_1_(1–62)-Y33W and BovIF_1_(1–62)-Y33W-H49K, without and with the mutation H49K, respectively. The eight versions of the human inhibitor protein included two versions of the full-length dimeric WT protein, HumIF_1_(1–81)-WT and HumIF_1_(1–81)-Y33W, plus the constitutively active forms HumIF_1_(1–81)-H49K and HumIF_1_(1–81)-Y33W-H49K. In addition, four forms of human IF_1_ were prepared to investigate the possible role of phosphorylation of residue Ser-14 in the regulation of the hydrolytic and synthetic activities of the protein, all with the mutation Y33W. The four versions, where Ser-14 has been replaced by the phosphomimetic residues aspartic acid and glutamic acid, are HumIF_1_(1–81)-S14D-Y33W, HumIF_1_(1–81)-S14D-Y33W-H49K, HumIF_1_(1–81)-S14E-Y33W and HumIF_1_(1–81)-S14E-Y33W-H49K. All fourteen proteins were produced by bacterial overexpression, with full-length bovine proteins prepared by a combination of cation and anion exchange chromatography and the other twelve by affinity purification and proteolytic removal of affinity tags. Their purities were validated by SDS-PAGE and their sequences were checked by MS (Figs. S2 and S3, and Table S4).

### Characterization of bovine SMPs

**Figure 2:**
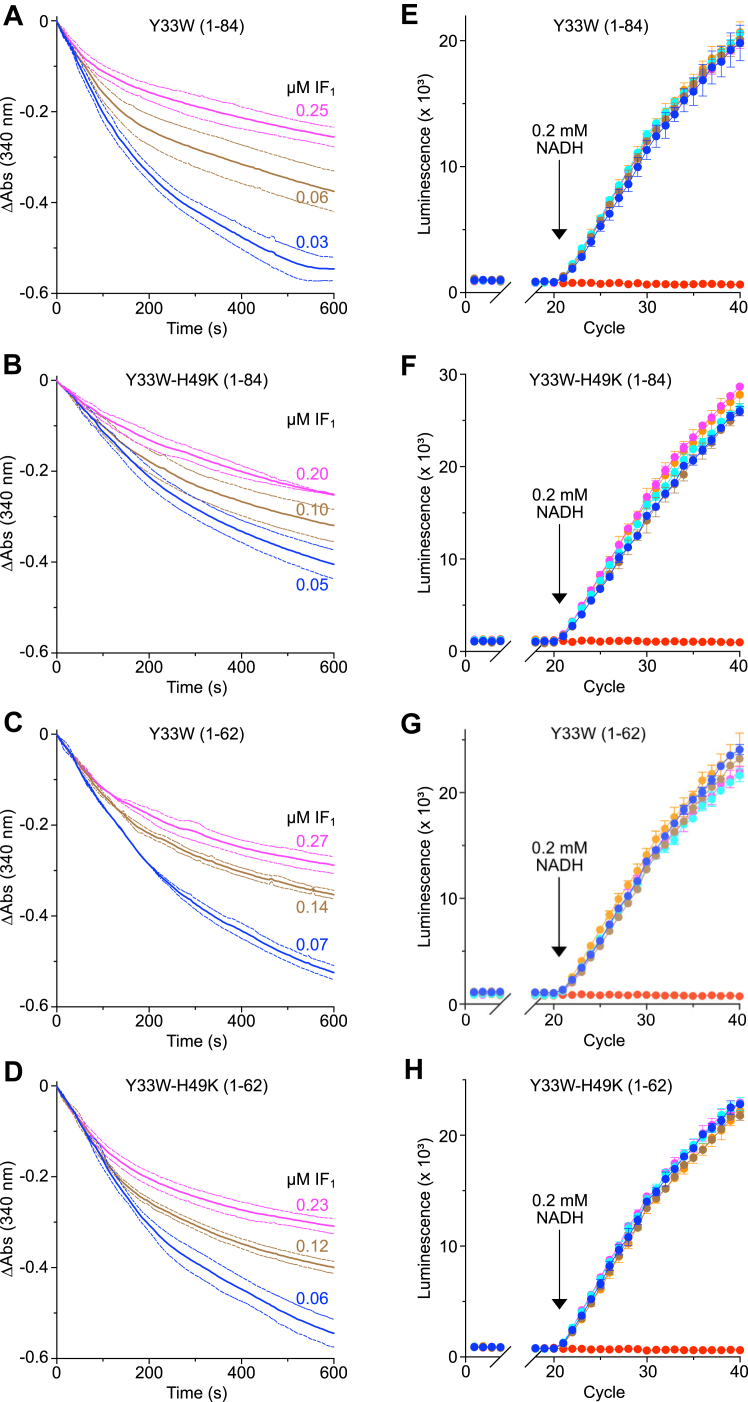
**Inhibition of ATP hydrolysis and the continued generation of ATP in the presence of mutated forms of bovine IF**_**1**_**.***A*–*D*, inhibition of ATP hydrolysis and (*E*–*H*), effects on ATP synthesis in bovine SMPs by increasing concentrations of mutant forms of bovine IF_1_. *A* and *E*, IF_1_(1–84)-Y33W, (*B* and *F*), IF_1_(1–84)-Y33W-H49K; (*C* and *G*), IF_1_(1–62)-Y33W; (*D* and *H*), IF_1_(1–62)-Y33W-H49K. *A*–*D*, measurements were made in triplicate, except for B 0.20 μM where duplicate values were used, average values were corrected to an initial value of zero, and ± SD shown with bounding *dashed lines* (Files S1–S4). In *E*–*H*, luminescence signals are given in relative light units; (), SMPs only; (), (), and (), SMPs in the presence of inhibitor proteins in molar ratios of 0.5, 2.5, 5, and 10, respectively, with respect to the quantity of ATP synthase; (), in the presence of oligomycin (10 μg/ml). Background luminescence levels were established for 20 measurement cycles, and ATP generation was initiated at cycle 20 by the addition of NADH (0.2 mM). Measurements were made in triplicate and average values ± SD are shown (Files S5-S8). SMP, submitochondrial particle.

ATP synthesis by bovine SMPs was demonstrated by generating a pmf with either NADH or succinate, in the presence of ADP and phosphate. This synthetic activity was abolished by the separate addition of the uncoupling agent carbonyl cyanide 4-(trifluoromethoxy)-phenylhydrazone and by oligomycin, rotenone, and antimycin, specific inhibitors of ATP synthase, complex I and complex III, respectively (Fig. S4). As expected, the addition of the inhibitors BovIF_1_(1–84)-Y33W, BovIF_1_(1–84)-Y33W-H49K, BovIF_1_(1–62)-Y33W and BovIF_1_(1–62)-Y33W-H49K, over a range of concentrations, had similar effects in inhibiting ATP hydrolysis (Fig. 2). In contrast, addition of the same proteins had no significant effect on ATP synthesis, whereas in control samples lacking the inhibitor protein, ATP synthesis was abolished by the addition of oligomycin (Fig. 2).

### Effects of human inhibitor proteins on the hydrolytic and synthetic activities of bovine ATP synthase

**Figure 3:**
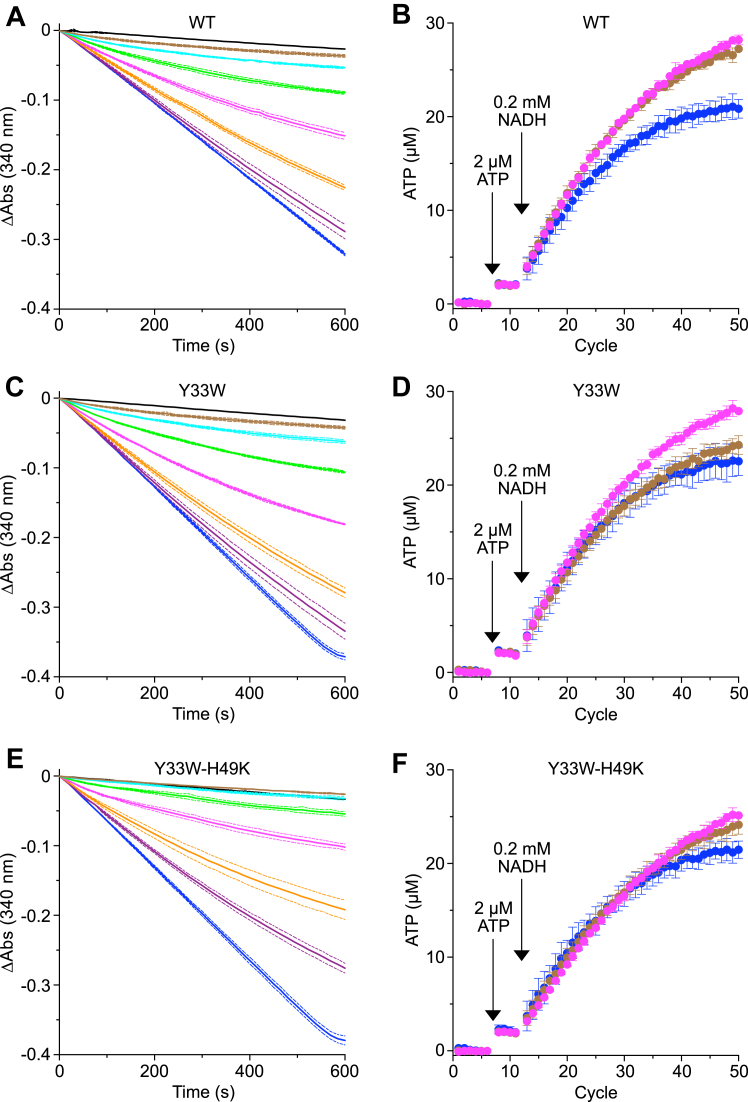
**Effect of human IF**_**1**_**and mutant forms on the activity of ATP synthase.***Left hand panels* (*A*), (*C*), and (*E*), ATP hydrolysis by bovine SMPs inhibited by increasing concentrations of (*A*), HumIF_1_(1–81); (*C*), HumIF_1_(1–81)-Y33W; (*E*), HumIF_1_(1–81)-Y33W-H49K. (), no inhibitor; (—), plus 1 μM oligomycin. IF_1_ concentrations: (), 0.05 μM; (), 0.1 μM; (), 0.2 μM; (), 0.4 μM; (), 0.8 μM; (), 1.6 μM. Measurements were made in triplicate, and the average values are shown, corrected to an initial value of zero, and ± SD shown as bounding *dashed lines* (Files S9–S11). *Right hand panels* (*B*), (*D*), and (*F*), ATP synthesis by bovine heart SMPs coupled to NADH oxidation, in the presence or absence of (*B*), HumIF_1_(1–81)-WT; (*D*), HumIF_1_(1–81)-Y33W; (*F*), HumIF_1_(1–81)-Y33W-H49K. ATP was measured by a luminescence continuous real-time assay with a luciferase-luciferin reagent. The luminescence signal was calibrated with 2 μM ATP, and the background signal before addition of ATP was subtracted. ATP synthesis was initiated by the addition of NADH (0.2 mM). (), no inhibitor; (), 0.5 μM IF_1_; (), 5 μM IF_1_. IF_1_ at 5 μM provides an IF_1_:ATP synthase molar ratio of 393:1. Data points are the average signal ± SD, n = 4 wells (Files S12–S14). SMP, submitochondrial particle.

**Figure 4:**
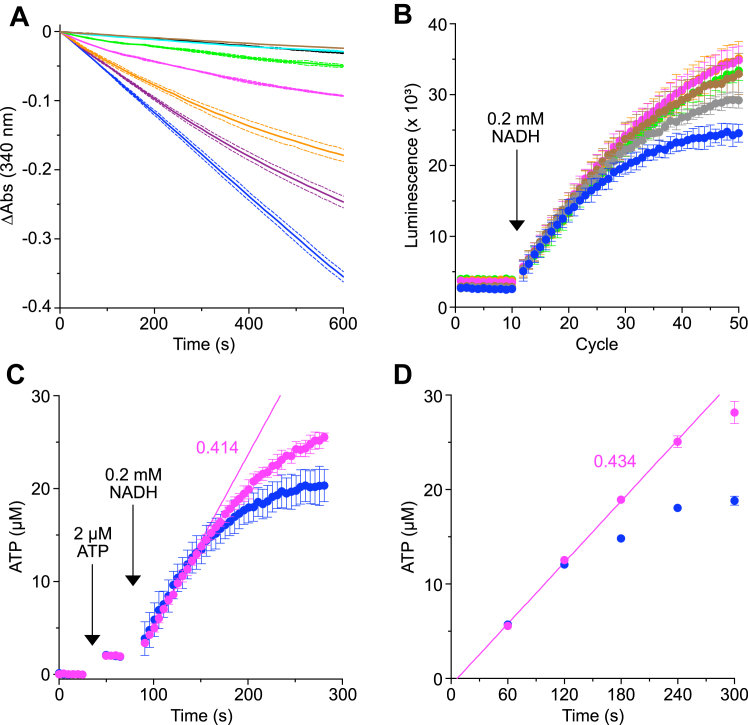
**Impact of constitutively active human IF**_**1**_**on ATP hydrolysis and ATP synthesis by bovine SMPs.***A*, ATP hydrolysis inhibited by increasing concentrations of constitutively active human IF_1_(1–81)-H49K. (), no inhibitor; (—), plus 1 μM oligomycin. IF_1_ at various concentrations; (), 0.05 μM; (), 0.1 μM; (), 0.2 μM; (), 0.4 μM; (), 0.8 μM; (), 1.6 μM. Measurements were made in triplicate, and the traces represent the average values corrected to an initial value of zero, and ± SD shown with bounding *dashed lines* (File S15). *B*–*D*, ATP synthesis by bovine SMPs coupled to NADH oxidation, in the presence or absence of human IF_1_(1–81)-H49K. ATP synthesis was initiated by the addition of NADH (0.2 mM). In (*B*) and (*C*), ATP synthesis was measured with a luminescence continuous real-time assay, and in (*D*), by a quench assay with luciferase-luciferin reagent. In (*B*), the concentrations of human IF_1_(1–81)-H49K were as follows: (), no inhibitor; (), 0.2 μM; (), 1 μM; (), 5 μM; (), 10 μM; (), 20 μM. A concentration of 20 μM IF_1_ provides a molar excess of ca. 1600:1 with respect to the quantity of ATP synthase. Background luminescence levels were established for ten measurement cycles. N = 4 wells, and data points correspond to the average signal ± SD (File S16). In (*C*), the luminescence signal was calibrated with 2 μM ATP before the addition of NADH, and the background signal before addition of ATP was subtracted. (), no inhibitor; (), 5 μM IF_1_(1–81)-H49K. The data points are the average signal ± SD, n = 4 wells (File S17). *D*, ATP synthesis determined with the quench assay. (), no inhibitor; (), 5 μM IF_1_(1–81)-H49K, corresponding to an IF_1_:ATP synthase molar ratio of ca. 700:1. Data points are the average signal ± SD, n = 3 (File S18). In (C), a linear regression () was applied to the initial rate (25 s) of ATP synthesis in the presence of IF_1_ H49K, and in (D) to the rate over 240 s. The rates of ATP synthesis (*magenta*) in μmol min^−1^ mg^−1^ were calculated from the slopes. SMP, submitochondrial particle.

The four inhibitor proteins HumIF_1_(1–81), HumIF_1_(1–81)-Y33W, HumIF_1_(1–81)-H49K, and HumIF_1_(1–81)-Y33W-H49K all inhibited ATP hydrolysis by bovine SMPs effectively. Constitutively active forms with the mutation H49K were the most potent, and at a concentration of 0.8 μM they inhibited hydrolysis as effectively as 1 μM oligomycin (Figs. 3 and 4). However, all four of these human inhibitor proteins had no significant effect on ATP synthesis (Figs. 3 and 4). Indeed, at the higher concentrations employed, the human inhibitor proteins had a stimulatory effect on ATP synthesis, presumably arising from the inhibition of the low levels of ATP hydrolysis by ATP synthase in a small fraction of leaky SMPs that are unable to maintain a pmf or possibly from uncoupled partially formed ATP synthase complexes that arise as intermediates during the process of assembly of the enzyme (19, 20). Similar stimulatory effects on ATP synthesis were especially evident, for example, in the presence of large molar excesses of BovIF_1_(1–84)-Y33W (Fig. S5). In the presence of HumIF_1_(1–81)-H49K, the rate of ATP synthesis calculated from the initial slope of the continuous assay was 0.414 μmol min^−1^ mg^−1^ bovine SMPs and 0.434 μmol min^−1^ mg^−1^ SMPs from the quench assay (Fig. 4). As the ATP synthase content of SMPs is 0.48 nmol mg^−1^ (Table S2), the calculated rate of synthesis of ATP approaches 15 molecules s^−1^ enzyme^−1^, which corresponds to the enzyme's rotor turning at ca. five revolutions per second, assuming that all ATP synthase complexes are active.

### Are the inhibitory properties of IF_1_ regulated by phosphorylation?

**Figure 5:**
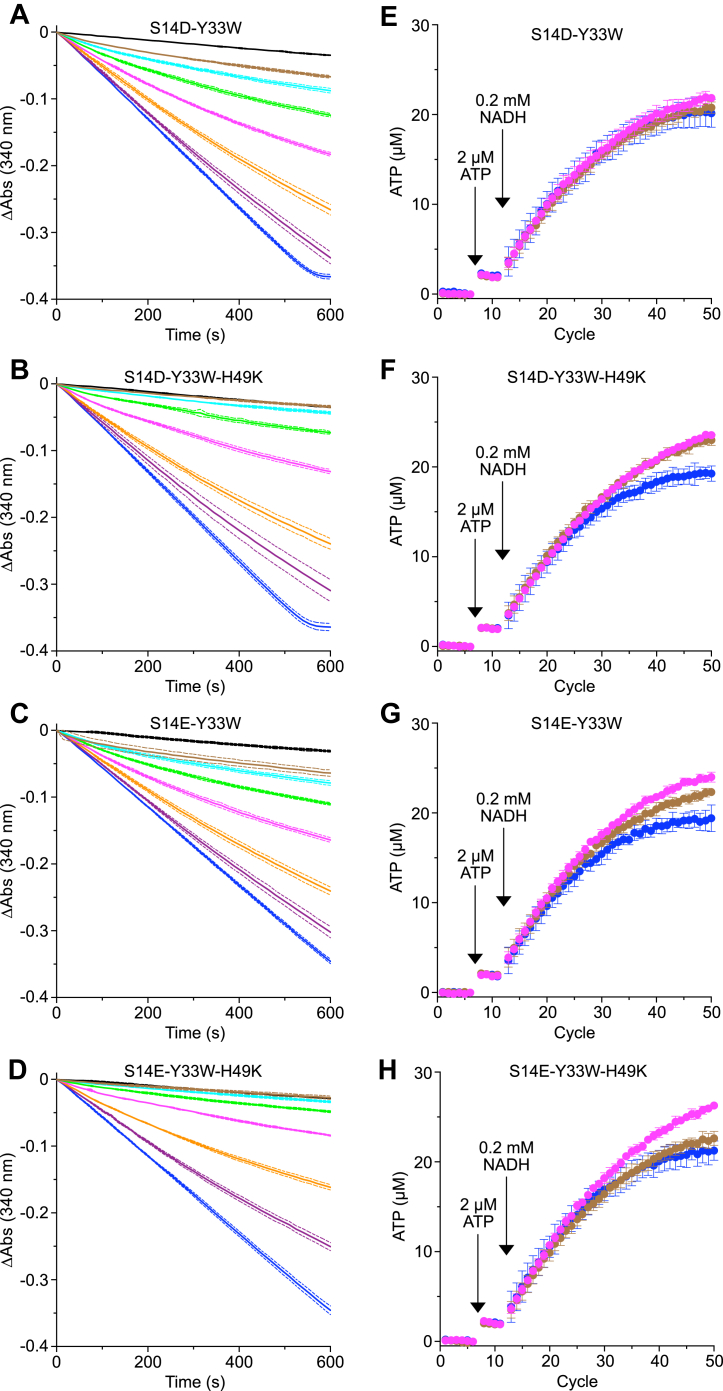
**Impact of phosphomimetic mutant forms of human IF**_**1**_**on the hydrolytic and synthetic activities of bovine ATP synthase.***A*–*D*, the impacts on ATP hydrolysis. *E*–*H*, the impacts on ATP synthesis. The following inhibitors were employed. In (*A*) and (*E*), IF_1_(1–81)-S14D-Y33W; in (*B*) and (*F*), IF_1_(1–81)-S14D-Y33W-H49K; in (*C*) and (*G*), IF_1_(1–81)-S14E-Y33W; in (*D*) and (*H*), IF_1_(1–81)-S14E-Y33W-H49K. In (*A*–*D*), inhibition of ATP hydrolysis by bovine SMPs by increasing concentrations of IF_1_. (), 0.05 μM; (), 0.1 μM; (), 0.2 μM; (), 0.4 μM; (), 0.8 μM; (), 1.6 μM. () and (—), controls with no inhibitor and 1 μM oligomycin, respectively. Measurements were made in triplicate, and traces correspond to the average values corrected to an initial value of zero, with ± SD shown as bounding *dashed lines* (Files S19–S22). In (*E*–*H*), ATP synthesis by bovine heart SMPs coupled to NADH oxidation, measured by a luminescence continuous real-time assay and with a luciferase-luciferin reagent. The luminescence signal was calibrated with 2 μM ATP, and the background signal before addition of ATP was subtracted. ATP synthesis was initiated by the addition of NADH (0.2 mM). (), no inhibitor; (), 0.5 μM IF_1_; (), 5 μM IF_1_. A concentration of 5 μM IF_1_ provides a molar ratio of ca. 400:1 with respect to the ATP synthase. Data points are the average signal ± SD, n = 4 wells (Files S23–S26). SMP, submitochondrial particle.

The proposal that the inhibitory activity of human IF_1_ is regulated by the phosphorylation of residue Ser-14 (13) was investigated with the four inhibitor proteins, HumIF_1_(1–81)-S14D-Y33W, HumIF_1_(1–81)-S14E-Y33W, and constitutively active forms HumIF_1_(1–81)-S14D-Y33W-H49K and HumIF_1_(1–81)-S14E-Y33W-H49K. All four were as effective inhibitors of ATP hydrolysis as the inhibitor protein containing Ser-14, and none of them inhibited ATP synthesis (Fig. 5). On this basis, it is concluded that the inhibitory activity of human IF_1_ on ATP hydrolysis is unlikely to be regulated by phosphorylation of Ser-14, and these experiments provide further evidence that human IF_1_ does not inhibit ATP synthesis.

## Discussion

### Fundamental properties of IF_1_

Three fundamental properties of the free bovine dimeric inhibitor that are likely to underlie its biological role in regulating the ATP synthase in both bovine and human mitochondria have been established previously by *in vitro* experimentation. The first such property is that the N-terminal portion of the inhibitory region from residue 1 to about residue 45 of bovine IF_1_ is intrinsically disordered (8, 9, 10). The N-terminal region of the human protein is predicted to be disordered also up to around residue 28, as it is also in the bovine protein (Fig. 1*C*), and, given that the disordered region of bovine IF_1_ has been demonstrated experimentally to extend up to around residue 45, the sequence conservation implies that it is likely to do so also in the human protein. It is noteworthy that a property of many intrinsically disordered proteins is that they bind to more than one biological target (21, 22). This characteristic raises the possibility that in addition to binding to the ATP synthase, IF_1_ binds to other as yet uncharacterized sites. This feature may help to explain some of the many diverse roles that have been connected to IF_1_ (see below).

A second fundamental property of mammalian IF_1_ is that the active inhibitor is dimeric, (see Fig. 1*A*). Previously, it has been demonstrated that each of the inhibitory regions in dimeric bovine IF_1_ can bind simultaneously to an F_1_-catalytic domain (23). However, the recent structure of the intact dimeric ATPase shows that the two F_1_-heads are too far apart to permit the dimeric inhibitor to straddle between the two catalytic domains in the isolated dimer (17). In the inner membranes of mitochondria, the dimers self-associate in long rows on the tips of the cristae (24, 25), and IF_1_ dimers can span across the dimer–dimer interface between adjacent F_1-_domains and such complexes have been observed by cryo-EM (26, 27). Although the biological significance of these inhibited complexes is currently unclear, they may participate in the formation of the variety of structures that the cristae can assume. Cells both overexpressing or lacking IF_1_ are reported to have altered cristae (28, 29, 30, 31).

A third fundamental property is that the inhibitory activity of bovine IF_1_ is influenced by both the pH and the ionic strength of cations of the surrounding milieu (6, 9). Thus, at around pH 8 and above, and at low concentrations of cations, the dimeric IF_1_ is inactive because the dimers self-associate and form oligomers of dimers where the inhibitory regions are occluded. At pH values of around 7 and below, and/or at high concentrations of cations, IF_1_ consists of free and active dimers, where the N-terminal inhibitory regions can bind to ATP synthase complexes and prevent the hydrolysis of ATP. This *in vitro* inactivation-activation process provides the basis for a possible mechanism of the physiological regulation of the activity of IF_1_. Thus, for example, the inactive inhibitor could be activated from inactive oligomers by a decrease in the pH of the mitochondrial matrix, such as occurs during ischemia (32). It could also be activated by an increased concentration of Ca^2+^ ions in the mitochondrial matrix either accompanying cell signalling events or by the cytoplasmic overload of Ca^2+^ ions and their subsequent uptake into the mitochondrial matrix, the opening of the mitochondrial permeability transition pore, disruption mitochondrial membranes, and necrotic cell death (33).

The experiments described here have confirmed and further defined a fourth fundamental property of IF_1_, namely that it is a unidirectional inhibitor of ATP hydrolysis only, with no direct effect on the synthesis of ATP. Kobayashi *et al.* (4) have shown that the physical restoration of rotation in the F_1_ domain from an IF_1_ inhibited state depended upon both the direction of application of the force and the nature of the bound substrate. Thus, it required both the manipulation of the γ-subunit in the synthetic direction and the presence of bound ADP and Pi before significant levels of rotation could be observed. These experiments also suggested that the re-establishment of rotation was accompanied by the release of bound IF_1_. Moreover, as shown here, there was no support for the proposal that the inhibitory activity of IF_1_ is regulated by phosphorylation of Ser-14 (13), as the replacement of this residue by either of the phosphomimetic residues aspartate or glutamate had no effect on the inhibitory properties of IF_1_. To a first approximation, the carboxylate of an aspartate or glutamate residue provides a point negative charge to mimic the point negative charge of a phosphoserine, but unlike a dianionic phosphate monoester, it has one negative charge only. Also, the aspartate and glutamate residues are smaller and have a different geometry from phosphoserine. Hence, although the introduction of an aspartate or a glutamate often mimics the function of phosphoserine; for examples see (34, 35), it may not necessarily do so (36). However, crucially, it should also be noted that Ser-14 of IF_1_ is not strictly conserved in mammals and other vertebrates (Fig. S6), as might be expected if the residue were involved in a basic regulatory mechanism. For example, in cattle, pigs, horses, African bush elephants, and bottle-nose dolphins it is replaced by an alanine residue (Fig. S6). Recent work also refutes the proposed inhibition of ATP synthesis by IF_1_ in cell cultures, and the regulation of IF_1_ by PKA-dependent phosphorylation (37). Therefore, on the basis of the experimental evidence and evolutionary considerations, on balance it is unlikely that the activity of mammalian IF_1_ is regulated *in vivo* by the reversible phosphorylation of Ser-14.

The structural basis of the unidirectionality of inhibition is not understood currently, but it could stem from two sources: the catalytic F_1_-domain, the inhibitor protein IF_1_, or both. While the structure of the catalytic domain of the enzyme in hydrolytic mode is well known (38, 39), there is no detailed structural information about the enzyme in synthetic mode. In synthetic mode, the rotor turns in a counter-clockwise sense (as viewed perpendicular to the plane of the mitochondrial membrane with the mitochondrial matrix above), and the torque of the rotor is driven by the pmf, whereas in hydrolytic mode, the rotor turns in the opposite sense and rotation is driven by the binding and release of substrates and products from the catalytic domain (38, 39). Thus, it seems possible, even likely, that the detailed structure of the enzyme will differ in these two modes, with perhaps the most significant differences being found in the structure of membrane extrinsic part of the rotor (made of single copies of the γ-, δ-, and ε-subunits) and especially of the elongated α-helical structure of the γ-subunit with which the N-terminal region of IF_1_ interacts in hydrolytic inhibition. Thus, the reversal of the direction of rotation with accompanying changes in the catalytic interfaces themselves can be envisaged to release any inhibitor bound previously in hydrolytic mode and prevent the inhibitor from (re)binding during synthetic mode. Removal of the unstructured residues 1 to 12 of IF_1_ did not affect the inhibitory activity significantly, but further truncation of IF_1_ up to residue 22 resulting in removal of the short α-helix and the N-terminal region of the long α-helix (Fig. 1) resulted in weaker binding of the truncated versions of IF_1_ to F_1_-ATPase (16), accompanied by a significant loss of the rotational and directional-dependent activation of hydrolytic activity (4).

### Roles of IF_1_

Many biological roles have been ascribed to IF_1_ (reviewed in ref (14)), and, for example, its roles as a factor in the process of assembly of ATP synthase clearly relate to its inhibition of partially formed intermediates that are capable of ATP hydrolysis but not synthesis (19, 20). Also, its association with dimeric ATP synthase complexes in influencing the formation of the mitochondrial cristae points to the local inhibition of ATP hydrolysis. In addition, it is accepted that following the dissipation of the mitochondrial pmf and lowered matrix pH associated with ischaemia that IF_1_ is a reversible noncompetitive inhibitor of ATP hydrolysis by mitochondrial ATP synthase. More recently it was shown that altering the levels of IF_1_ affects cellular and mitochondrial Ca^2+^ handling (40) and may be unrelated to the interaction of IF_1_ with ATP synthase (41). Perhaps the most widely discussed and least understood role is the association of IF_1_ with various forms of cancer (14, 42). It has been observed that IF_1_ is expressed at elevated levels in many forms of the disease, and it has been suggested to play a role in the switch of the cancer cells to a more aerobic glycolytic metabolism (43, 44, 45). However, the molecular mechanisms underlying this pro-oncogenic role remain obscure. These suggestions that IF_1_ inhibits ATP synthesis are based on indirect observations, and not, as here, by direct biochemical analysis. It remains possible that the intrinsically disordered region of IF_1_ leads it to bind not only to the mitochondrial ATPase but also to other, as yet unidentified, targets, and that this property may provide an explanation of the apparently contradictory suggestions that have been made about the properties of the protein.

## Experimental procedures

### Numbering of IF_1_ proteins

The 25 amino acid N-terminal mitochondrial import sequences of bovine and human IF_1_ are removed during import of the proteins into the organelle, and the mature proteins are numbered from 1 to 84 and 1 to 81, respectively. In mitochondria, both mature proteins have a ragged N-terminus with forms starting at residues −1, 1, 2, and 3 (46, 47, 48).

### Protein estimation

The concentration of proteins in solution were determined with bicinchoninic acid (BCA, Thermo Fisher Scientific). In experiments with bovine IF_1_ containing the mutation Y33W, protein concentrations were estimated from the UV absorbance at 280 nm.

### Bovine SMPs

Bovine mitochondria from approximately half a heart, prepared as described before (49), were resuspended in SMP buffer (10 mM 3-(N-Morpholino)propanesulphonic acid, pH 7.5, and 250 mM sucrose) and centrifuged (11,300*g*, 12 min, 4 °C). The pellets were resuspended in 30 ml of SMP buffer with a Dounce homogenizer. The pH of the suspension was adjusted to 9 with 2.5 M Tris base, stirred at 4 °C for 10 min, and then diluted with SMP buffer (30 ml), and centrifuged (38,000*g*, 14 min, 4 °C). The pellet was resuspended in SMP buffer (20 ml), and the washing procedure was repeated twice more. The suspension of washed mitochondria in 40 ml of SMP buffer, plus bovine cytochrome c (final concentration 100 μM) was kept at 4 °C for 1 h. Magnesium sulfate was added (final concentration 15 mM), and the suspension was sonicated ten times at 150 W for 15 s at 4 °C, with 1 min intervening pauses, with a Q700 sonicator (QSonica; probe diameter 0.5 inches). Then the suspension was centrifuged (24,700*g*, 20 min), and the supernatant was recentrifuged (74,700*g*, 30 min, 4 °C). The resultant pellet of SMPs was resuspended in SMP buffer, homogenized, and stored at −80 °C.

### ATP synthase content of SMPs

Bovine SMPs (41 mg/ml) were diluted to 10 mg/ml and extracted for 15 min at 4 °C with buffer containing 0.1 M ammonium bicarbonate, pH 8, 0.2 mM neutral tris(2-carboxyethyl)-phosphine) (TCEP, Sigma), and 1% (w/v) *n*-dodecyl-β-D-maltoside (final concentration). The sample was centrifuged (14,000*g*, 15 min, 4 °C), and proteins in the supernatant at a concentration of 1 mg/ml, estimated with BCA, were digested for 2 to 2.5 h at 37 °C with trypsin (sequencing grade from Roche; trypsin:protein, 1:100, w/w) in the presence of 0.1 M ammonium bicarbonate, 0.2 mM TCEP, and 0.5% (w/v) sodium deoxycholate (50). Then digestion was continued for a further 18 h at 37 °C in the presence of a second portion of trypsin and finally in the presence of a third portion of trypsin for 1.5 to 2 h. The concentrations of synthetic peptides containing stable isotopes (Table S5; Cambridge Research Biochemicals) dissolved in 10% (v/v) aqueous acetonitrile were determined by amino acid analysis (Protein and nucleic acid facility, Department of Biochemistry, University of Cambridge). A known amount of this internal reference standard was added to the digest (51), plus an equal volume of ethyl acetate saturated with water and containing formic acid (ca. 4%, v/v) (50). The sample was vortexed, then centrifuged (14,000*g*, 2 min, 18 °C) to separate the phases. Portions of the lower aqueous phase containing the peptides was fractionated on a C_18_ reverse phase column in a Proxeon EASY-nLC1000, eluted with a gradient of acetonitrile containing 0.1% (v/v) formic acid, with the column effluent coupled directly to a Q-Exactive Orbitrap mass spectrometer (Thermo Fisher Scientific). Peptides were fragmented by collision-induced dissociation with nitrogen. Peak areas for the labeled and endogenous peptides (charge state 2^+^) were calculated from the extracted ion chromatograms of the parent ions with Xcalibur (Thermo Fisher Scientific) with an *m/z* tolerance of 5 ppm, and used to determine the subunit ratio and absolute amount of ATP synthase (Fig. S7, Table S6). Labeled peptides gave a linear signal over the analytical range of 31.3 to 2000 fmol (Fig. S8).

### Overexpression of WT and mutant forms of bovine and human IF_1_

Bovine and human IF_1_ variants were constructed with the mutation Y33W to facilitate protein quantitation by absorbance at 280 nm and used as a calibration standard for protein estimations by the BCA method. The mutation H49K was introduced into the bovine protein by site directed mutagenesis with the forward primer agaaacac**aag**gaaaatgagatctctcatcatgcaaaggag and the reverse primer tcattttc**ctt**gtgtttcttcaaggcggccagctgttctttag, where the bold letters indicate the mutated codon. The primers were designed to anneal to the same sequence on the opposite strands of the plasmid. The entire plasmid was amplified by PCR, and template DNA was digested with the restriction enzyme *Dpn I*. The sequences were cloned into the expression vector pRun and were confirmed by DNA sequencing. The DNA sequence for human IF_1_ encoded the intact protein (residues 1–81) plus the N-terminal sequence MSHHHHHHSAENLYFQ. Specific mutations were introduced into the coding sequence of the human protein with the NEBaseChanger kit (New England Biolabs). Expression plasmids encoding WT and mutated forms of bovine and human IF_1_ were transformed into cells of *Escherichia coli* C41 (DE3). Cells were grown in 2xTY medium at 37 °C until the *A*_600_ reached 0.6 absorbance units. Then expression of recombinant proteins was induced with IPTG (final concentration 600 μM). Cells expressing bovine proteins were grown at 25 °C for 18 h, centrifuged (6500*g*, 10 min), resuspended in buffer containing 10 mM Tris–HCl, pH 8, 1 mM EDTA, and complete EDTA-free protease inhibitors (Sigma-Aldrich), and broken with a Constant Cell disruptor (Constant Cell). Subsequent steps were carried out at 4 °C. Broken cells were centrifuged (265,000*g*, 45 min), and the supernatant was dialyzed for 18 h against buffer A (10 mM Tris–HCl, pH 8, and 1 mM EDTA). The solution was applied to a HiTrap SP HP cation exchange column (5 ml; GE Healthcare). The inhibitor protein eluted from the column at a salt concentration of 350 mM on a linear gradient of NaCl from 0 to 1 M. Fractions containing the inhibitor were dialyzed against buffer A for 18 h and then applied to a HiTrap Q HP column (5 ml; GE Healthcare). On a linear gradient of NaCl from 0 to 1 M, the inhibitor protein eluted at 300 mM. Purified IF_1_ was exchanged into ultrapure water on a PD-10 column (GE Healthcare). Cells expressing human IF_1_ and variants were grown at 37 °C for ca. 4 h, centrifuged (2700*g*, 10 min, 4 °C), washed in cold buffer A consisting of 20 mM Tris–HCl, pH 7.4, 0.1 M NaCl, 10% (v/v) glycerol and containing cOmplete protease inhibitors (Sigma-Aldrich), and centrifuged (3900*g*, 10 min, 4 °C). Cells were resuspended in cold buffer A containing cOmplete EDTA-free protease inhibitors and broken, on ice, with a Q700 sonicator equipped with sonic probe 0.75 inches in diameter. Broken cells were centrifuged (10,845*g*, 15 min, 4 °C), and imidazole (5 M, pH 8) was added to the supernatant to give a final concentration of 20 mM. The sample was mixed by rotation at 4 °C for 1 h with nickel-nitrilotriacetic acid agarose resin (Qiagen). Then the resin with IF_1_ bound *via* the His-tag was collected by settling, or centrifugation (27*g*, 1 min, 4 °C), washed with buffer A plus 20 mM imidazole, and finally IF_1_ was eluted with buffer A containing 0.5 M imidazole. The eluted IF_1_ was dialyzed in a 3.5 kDa cut-off Slide-A-Lyzer cassette (Thermo Fisher Scientific) into buffer A at 4 °C. The His-tag was removed in the dialysis cassette by addition of tobacco etch virus (TEV) protease (ca. 0.1 mg/ml, final concentration) and neutralized 2 mM TCEP and incubation with stirring at 30 °C for 3 h in buffer A. The cleaved sample was then kept for 1 h at 4 °C in the presence of the nickel-nitrilotriacetic acid agarose and 10 mM imidazole. The unbound IF_1_ was dialyzed at 4 °C against Milli-Q water in a 3.5 kDa cut-off Slide-A-Lyzer cassette.

### Overexpression and purification of C terminally truncated bovine IF_1_

DNA-encoding residues 1 to 62 of bovine IF_1_ with an N-terminal hexa-histidine tag, followed by a site for TEV protease, as described above for human IF_1_, was cloned into pRun. The point mutation H49K was introduced by site directed mutagenesis and the protein was expressed as described above for intact bovine IF_1_. The bacterial pellet was resuspended in His buffer A (20 mM Tris, pH 7.4, 0.1 M NaCl, and 10 mM imidazole) plus one cOmplete EDTA-free protease inhibitor tablet (Roche). Cells were broken with a constant cell disruptor and then centrifuged (234,000*g*, 45 min, 4 °C). The supernatant was applied to a HisTrap column (5 ml, GE Healthcare), which was eluted with a gradient of imidazole from 10 to 500 mM. The purified protein was dialyzed against His buffer A plus TCEP in the presence of TEV protease for 18 h at 18 °C to cleave the His-tag. The free His-tag was removed by affinity chromatography and the truncated forms were exchanged into ultrapure water.

### Characterization of inhibitor proteins

The purity of inhibitor proteins was examined by SDS-PAGE and staining with Coomassie R250. Stained gels were imaged with an Epson Perfection V850 Pro flatbed scanner. Intact molecular masses were measured by electrospray MS in a Q-Trap 4000 (ABSciex) instrument, operated in MS mode, and calibrated with a mixture of myoglobin and trypsinogen. Reconstructed molecular masses were calculated with Peakview (Sciex).

### Inhibition of ATP hydrolysis by SMPs

The ATP hydrolase activity of SMPs was measured in the presence of various inhibitor proteins with a coupled ATP-generating system (52). The inhibition of the ATP hydrolysis by endogenous ADP bound to SMPs was alleviated by incubation for 10 min at room temperature with an equal volume of stripping buffer (20 mM Tris–HCl, pH 7.4, 50% glycerol v/v, and 4 mM EDTA). The stripped SMPs were added to the assay mix consisting of 50 mM Tris–HCl, pH 7.4, 50 mM KCl, 2 mM MgCl_2_, 0.14 or 0.2 mM NADH, 1 μM rotenone or piericidin A, pyruvate kinase (20 μg/ml), and lactate dehydrogenase (10 μg/ml), to a final concentration of ca. 4 to 20 μg/ml, depending on the measured uninhibited activity. The assay mix (180 μl, 37 °C) was dispensed into a 96-well plate (Corning) warmed to 37 °C. The assay was initiated by the addition of 20 μl of a solution containing 10 mM phosphoenolpyruvate, 20 mM ATP, and the requisite inhibitor protein to give a final assay volume of 200 μl. The oxidation of NADH was monitored from the UV absorbance at 340 nm for 10 min at 37 °C with a SpectraMax M2 plate reader (Molecular Devices). Measurements were made in triplicate.

### ATP synthesis by SMPs

In some experiments, the synthesis of ATP was followed by a continuous real-time assay (53) and in others by a quench assay. In both assays, bovine SMPs were added to the buffer consisting of 20 mM Tris-phosphate, pH 7.4, and 5 mM MgCl_2_, plus 20 or 100 μM of the adenylate kinase inhibitor, P1,P5-di(adenosine-5′)pentaphosphate, and 100 μM ADP. The reaction mixture was warmed to 37 °C. The final concentration of ATP synthase in the assay solution was 0.5 to 1.5 pmol per 100 μl.

#### The continuous real-time assay

The luciferase reagent from ATP bioluminescence kit CLS II (Roche) was prepared according to the manufacturer’s instructions, and 1 μl was added to each 100 μl of assay solution containing the bovine SMPs dispensed in wells in opaque white 96-well plates (Corning or PerkinElmer) at 37 °C. Luminescence was measured with a ClarioStar plate reader (BMG Labtech). Typically, a single row of 12 wells was read simultaneously, with three samples in quadruplicate, distributed in a repeating pattern from the outside to the centre to help offset the time difference across the row. Background luminescence values were established over 10 to 20 measurement cycles (5 or 7 s with a measurement interval time of 0.1 s). Then a pmf was generated by the addition of either 200 μM NADH or 4 mM succinate, and the synthesis of ATP was followed during a further 20 to 40 cycles. In assays conducted in the presence of bovine and human IF_1_ and mutants thereof, the inhibitor proteins were preincubated with SMPs in the assay mixture for 2 to 3 min before initiating ATP synthesis by the generation of a pmf. Control assays were performed in the presence of the respiratory inhibitors oligomycin, carbonyl cyanide-p-trifluoromethoxyphenylhydrazone, rotenone, or antimycin A. Measurements were made in triplicate or quadruplicate.

#### The quench assay

The assay was performed in microcentrifuge tubes instead of in the wells of 96-well plates, in the absence of the luciferase reagent, but otherwise under the same conditions as described above. Samples (10 μl) were taken from the reaction mixture at 1 min intervals and added to 4% (w/v) trichloroacetic acid (40 μl), and mixed 20 s later with 1 M Tris–HCl, pH 8 (250 μl), at room temperature. The luciferase reagent (30 μl) was added to the mixture and three portions (100 μl) were transferred into wells in an opaque white 96-well plate. The luminescence signal was measured in a ClarioStar plate reader at room temperature. The instrument was calibrated with standard samples of ATP.

## Data availability

All data are available within the paper and Supporting information, except for MS data deposited to the ProteomeXchange Consortium *via* the PRIDE (54) partner repository with the dataset identifier PXD045079.

## Supporting information

This article contains supporting information (10, 55, 56).

## Conflict of interest

The authors declare that they have no conflicts of interest with the contents of this article.
