# Supplementary material for: The inhibitor protein IF1 from mammalian mitochondria inhibits ATP hydrolysis but not ATP synthesis by the ATP synthase complex
Source: J Biol Chem. 2024 Jan 26;300(3):105690. doi: 10.1016/j.jbc.2024.105690 (PMC10906535; doi:10.1016/j.jbc.2024.105690)
Supplement: Supporting Information [file mmc1.pdf]

Supporting Information for

The inhibitor protein IF_1 from mammalian mitochondria inhibits ATP hydrolysis but not ATP synthesis by the ATP synthase complex

Joe Carroll, Ian N. Watt, Charlotte Wright, Shujing Ding, Ian M. Fearnley and John E. Walker

The Medical Research Council Mitochondrial Biology Unit, University of Cambridge, Cambridge Biomedical Campus, Hills Road, Cambridge CB2 0XY, United Kingdom

Corresponding author: John E. Walker

Email: walker@mrc-mbu.cam.ac.uk

Supporting information included:

Figures S1-S8

Tables S1-S6

A

```
Bovine      QKTGTAEVSSILEERILGADTSVDLEETGRVLSIGDGIARVHGLRNVQAEEMVEFSSGLK   60
Human       QKTGTAEMSSILEERILGADTSVDLEETGRVLSIGDGIARVHGLRNVQAEEMVEFSSGLK   60
            *******:****************************************************

Bovine      GMSLNLEPDNVGVVVFGNDKLIKEGDIVKRTGAIVDVPVGEELLGRVVDALGNAIDGKGP   120
Human       GMSLNLEPDNVGVVVFGNDKLIKEGDIVKRTGAIVDVPVGEELLGRVVDALGNAIDGKGP   120
            ************************************************************

Bovine      IGSKARRRVGLKAPGIIPRISVREPMQTGIKAVDSLVPIGRGQRELIIGDRQTGKTSIAI   180
Human       IGSKTRRRVGLKAPGIIPRISVREPMQTGIKAVDSLVPIGRGQRELIIGDRQTGKTSIAI   180
            ****:*******************************************************

Bovine      DTIINQKRFNDGTDEKKKLYCIYVAIGQKRSTVAQLVKRLTDADAMKYTIVVSATASDAA   240
Human       DTIINQKRFNDGSDEKKKLYCIYVAIGQKRSTVAQLVKRLTDADAMKYTIVVSATASDAA   240
            ************:***********************************************

Bovine      PLQYLAPYSGCSMGEYFRDNGKHALIIYDDLSKQAVAYRQMSLLLRRPPGREAYPGDVFY   300
Human       PLQYLAPYSGCSMGEYFRDNGKHALIIYDDLSKQAVAYRQMSLLLRRPPGREAYPGDVFY   300
            ************************************************************

Bovine      LHSRLLERAAKMNDAFGGGSLTALPVIETQAGDVSAYIPTNVISITDGQIFLETELFYKG   360
Human       LHSRLLERAAKMNDAFGGGSLTALPVIETQAGDVSAYIPTNVISITDGQIFLETELFYKG   360
            ************************************************************

Bovine      IRPAINVGLSVSRVGSAAQTRAMKQVAGTMKLELAQYREVAAFAQFGSDLDAATQQLLSR   420
Human       IRPAINVGLSVSRVGSAAQTRAMKQVAGTMKLELAQYREVAAFAQFGSDLDAATQQLLSR   420
            ************************************************************

Bovine      GVRLTELLKQGQYSPMAIEEQVAVIYAGVRGYLDKLEPSKITKFENAFLSHVISQHQALL   480
Human       GVRLTELLKQGQYSPMAIEEQVAVIYAGVRGYLDKLEPSKITKFENAFLSHVVSQHQALL   480
            ****************************************************:*******

Bovine      SKIRTDGKISEESDAKLKEIVTNFLAGFEA   510
Human       GTIRADGKISEQSDAKLKEIVTNFLAGFEA   510
            ..**:******:******************
```

B

```
Bovine      AAQASPSPKAGATTGRIVAVIGAVVDVQFDEGLPPILNALEVQGRETRLVLEVAQHLGES   60
Human       AAQTSPSPKAGAATGRIVAVIGAVVDVQFDEGLPPILNALEVQGRETRLVLEVAQHLGES   60
            ***:********:***********************************************

Bovine      TVRTIAMDGTEGLVRGQKVLDSGAPIRIPVGPETLGRIMNVIGEPIDERGPIKTKQFAAI   120
Human       TVRTIAMDGTEGLVRGQKVLDSGAPIKIPVGPETLGRIMNVIGEPIDERGPIKTKQFAPI   120
            **************************:******************************* *

Bovine      HAEAPEFVEMSVEQEILVTGIKVVDLLAPYAKGGKIGLFGGAGVGKTVLIMELINNVAKA   180
Human       HAEAPEFMEMSVEQEILVTGIKVVDLLAPYAKGGKIGLFGGAGVGKTVLIMELINNVAKA   180
            *******:****************************************************

Bovine      HGGYSVFAGVGERTREGNDLYHEMIESGVINLKDATSKVALVYGQMNEPPGARARVALTG   240
Human       HGGYSVFAGVGERTREGNDLYHEMIESGVINLKDATSKVALVYGQMNEPPGARARVALTG   240
            ************************************************************

Bovine      LTVAEYFRDQEGQDVLLFIDNIFRFTQAGSEVSALLGRIPSAVGYQPTLATDMGTMQERI   300
Human       LTVAEYFRDQEGQDVLLFIDNIFRFTQAGSEVSALLGRIPSAVGYQPTLATDMGTMQERI   300
            ************************************************************

Bovine      TTTKKGSITSVQAIYVPADDLTDPAPATTFAHLDATTVLSRAIAELGIYPAVDPLDSTSR   360
Human       TTTKKGSITSVQAIYVPADDLTDPAPATTFAHLDATTVLSRAIAELGIYPAVDPLDSTSR   360
            ************************************************************

Bovine      IMDPNIVGSEHYDVARGVQKILQDYKSLQDIIAILGMDELSEEDKLTVSRARKIQRFLSQ   420
Human       IMDPNIVGSEHYDVARGVQKILQDYKSLQDIIAILGMDELSEEDKLTVSRARKIQRFLSQ   420
            ************************************************************

Bovine      PFQVAEVFTGHLGKLVPLKETIKGFQQILAGEYDHLPEQAFYMVGPIEEAVAKADKLAEE   480
Human       PFQVAEVFTGHMGKLVPLKETIKGFQQILAGEYDHLPEQAFYMVGPIEEAVAKADKLAEE   480
            ***********:************************************************

Bovine      HS-         482
Human       HSS         483
            **
```

C

```
Bovine          ATLKDITRRLKSIKNIQKITKSMKMVAAAKYARAERELKPARVYGVGSLALYEKADIKTP    60
Human           ATLKDITRRLKSIKNIQKITKSMKMVAAAKYARAERELKPARIYGLGSLALYEKADIKGP    60
                ******************************************:**:************ *

Bovine          EDKKKHLIIGVSSDRGLCGAIHSSVAKQMKSEAANLAAAGKEVKIIGVGDKIRSILHRTH    120
Human           EDKKKHLLIGVSSDRGLCGAIHSSIAKQMKSEVATLTAAGKEVMLVGIGDKIRGILYRTH    120
                *******:****************:*******.*.*:****** ::*:*****.**:***

Bovine          SDQFLVTFKEVGRRPPTFGDASVIALELLNSGYEFDEGSIIFNRFRSVISYKTEEKPIFS    180
Human           SDQFLVAFKEVGRKPPTFGDASVIALELLNSGYEFDEGSIIFNKFRSVISYKTEEKPIFS    180
                ******:******:*****************************:****************

Bovine          LDTISSAESMSIYDDIDADVLRNYQEYSLANIIYYSLKESTTSEQSARMTAMDNASKNAS    240
Human           LNTVASADSMSIYDDIDADVLQNYQEYNLANIIYYSLKESTTSEQSARMTAMDNASKNAS    240
                *:*::**:*************:*****.********************************

Bovine          EMIDKLTLTFNRTRQAVITKELIEIISGAAALD       273
Human           EMIDKLTLTFNRTRQAVITKELIEIISGAAALD       273
                *********************************
```

Fig. S1. Comparison of the sequences of the α-, β- and γ-subunits of bovine and human ATP synthase. The mature subunits are numbered from residue 1 to their C-termini. Symbols *, : and . denote identical, strongly conserved and weakly conserved residues respectively. (*A*) α-subunits (bovine, sp|P19483; human, sp|P25705) identical in 98.4% residues. (*B*) β-subunits (bovine, sp|P00829; human, sp|P06576), identical in 98.6% residues. (*C*) γ-subunits (bovine, sp|P05631; human, sp|P36542), identical in 92.3% residues with a further 6.2% residues highly conserved.

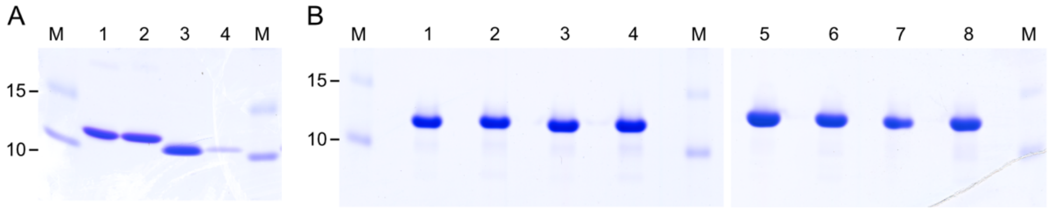

Fig. S2. Analysis of purified bovine and human IF$_1$ by SDS-PAGE. (*A*) Bovine IF$_1$. Lanes 1 and 2, bovine IF$_1$(1-84) with the mutations Y33W and Y33W-H49K, respectively; lanes 3 and 4, bovine IF$_1$(1-62) with the mutations Y33W and Y33W-H49K, respectively. (*B*) Human IF$_1$ (1-81). Lane 1, wild type (residues 1-81); 2-8, forms containing, respectively, the following mutations: 2, H49K; 3, Y33W; 4, Y33W-H49K; 5, S14D-Y33W; 6, S14D-Y33W-H49; 7, S14E-Y33W; 8, S14E-Y33W-H49K. M, molecular weight markers (kDa) are indicated.

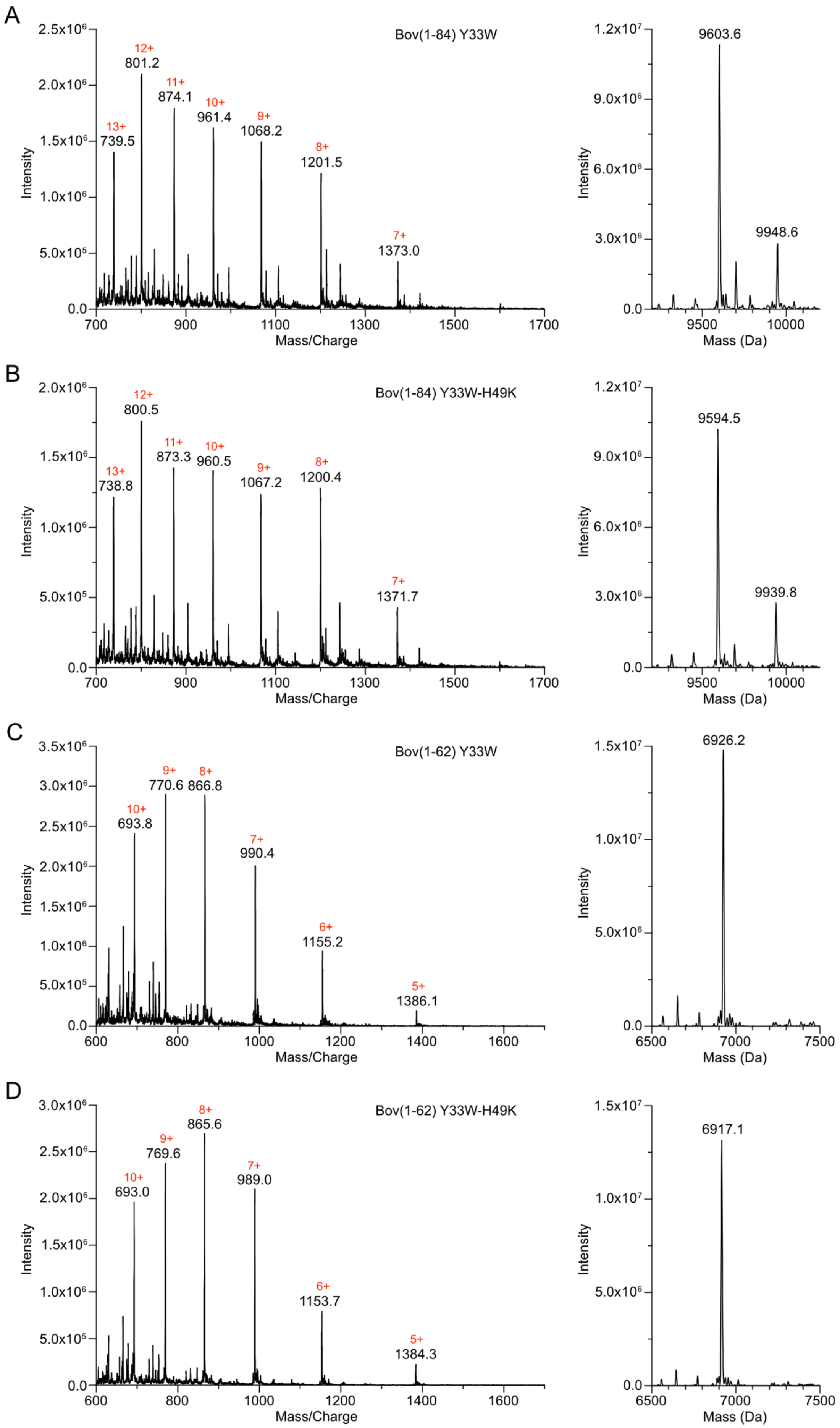
A
Bov(1-84) Y33W
Intensity
Mass/Charge
13+ 739.5
12+ 801.2
11+ 874.1
10+ 961.4
9+ 1068.2
8+ 1201.5
7+ 1373.0
9603.6
9948.6
Mass (Da)
B
Bov(1-84) Y33W-H49K
13+ 738.8
12+ 800.5
11+ 873.3
10+ 960.5
9+ 1067.2
8+ 1200.4
7+ 1371.7
9594.5
9939.8
C
Bov(1-62) Y33W
10+ 693.8
9+ 770.6
8+ 866.8
7+ 990.4
6+ 1155.2
5+ 1386.1
6926.2
D
Bov(1-62) Y33W-H49K
10+ 693.0
9+ 769.6
8+ 865.6
7+ 989.0
6+ 1153.7
5+ 1384.3
6917.1

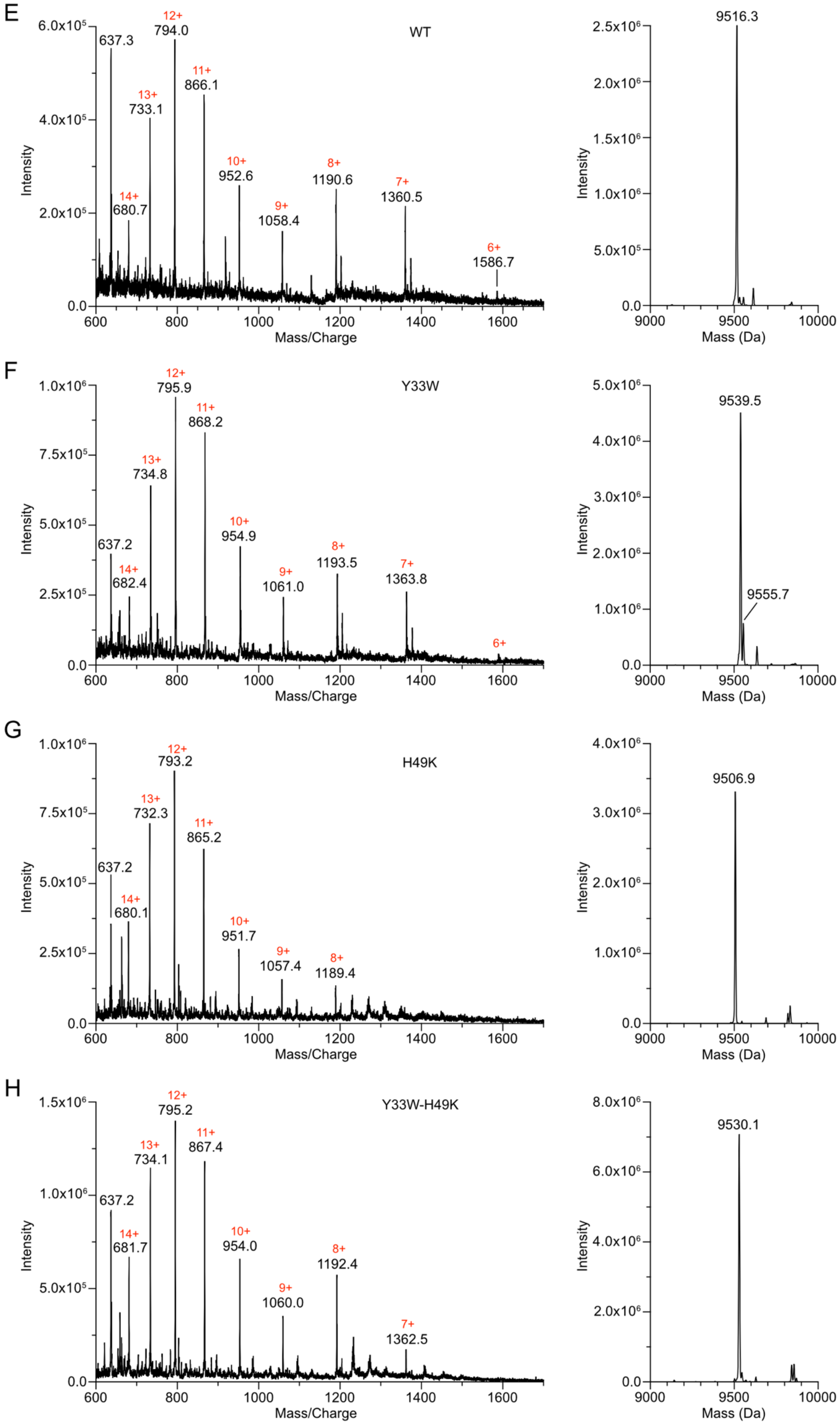
E
WT
Intensity
Mass/Charge
12+ 794.0
637.3
11+ 866.1
13+ 733.1
10+ 952.6
8+ 1190.6
7+ 1360.5
14+ 680.7
9+ 1058.4
6+ 1586.7
9516.3
Mass (Da)
F
Y33W
12+ 795.9
11+ 868.2
13+ 734.8
10+ 954.9
637.2
8+ 1193.5
7+ 1363.8
14+ 682.4
9+ 1061.0
6+
9539.5
9555.7
G
H49K
12+ 793.2
13+ 732.3
11+ 865.2
637.2
14+ 680.1
10+ 951.7
9+ 1057.4
8+ 1189.4
9506.9
H
Y33W-H49K
12+ 795.2
11+ 867.4
13+ 734.1
637.2
14+ 681.7
10+ 954.0
8+ 1192.4
9+ 1060.0
7+ 1362.5
9530.1

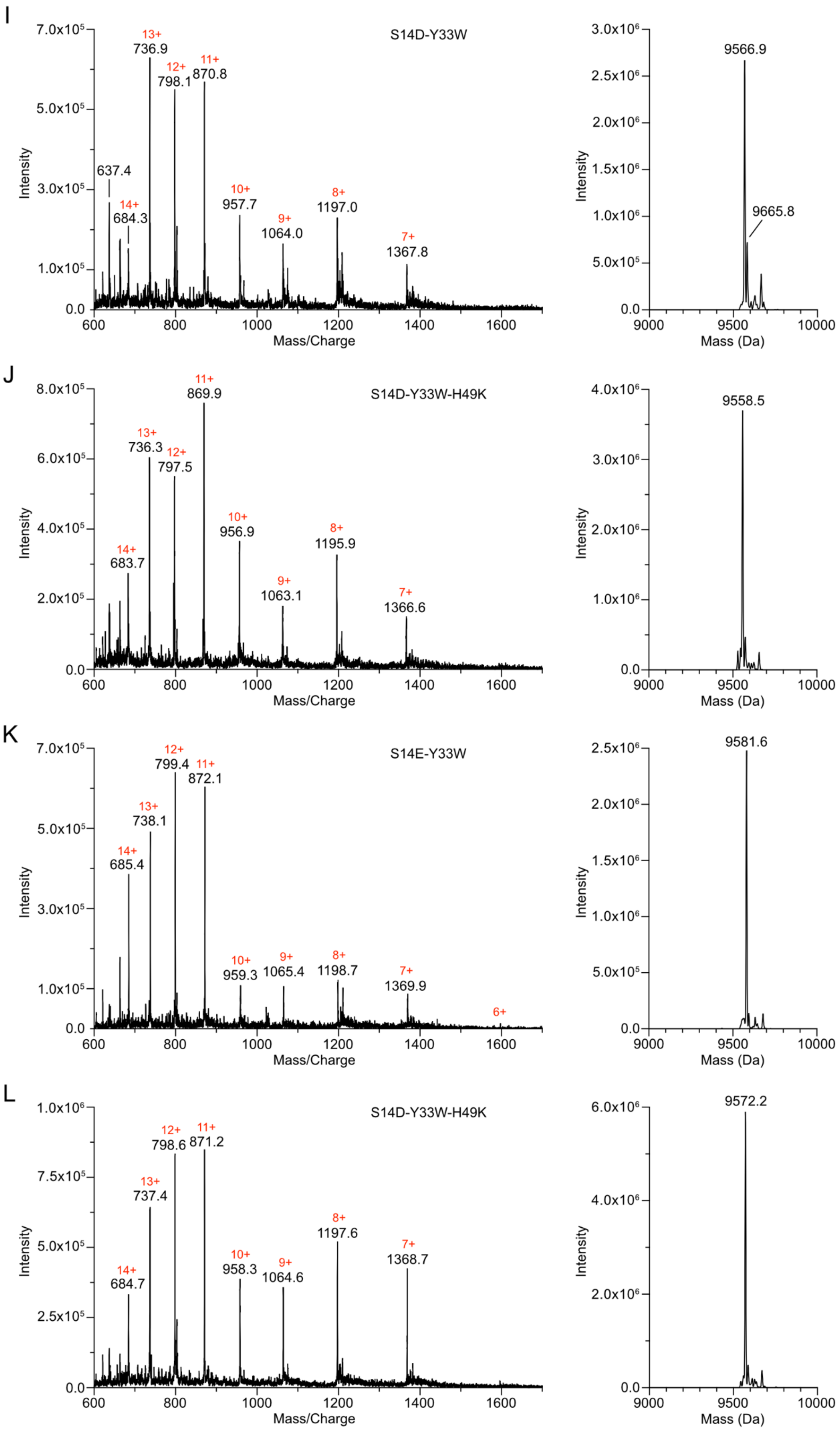
I
S14D-Y33W
Intensity
Mass/Charge
13+ 736.9
12+ 798.1
11+ 870.8
637.4
14+ 684.3
10+ 957.7
9+ 1064.0
8+ 1197.0
7+ 1367.8
9566.9
9665.8
Mass (Da)
J
S14D-Y33W-H49K
11+ 869.9
13+ 736.3
12+ 797.5
10+ 956.9
14+ 683.7
8+ 1195.9
9+ 1063.1
7+ 1366.6
9558.5
K
S14E-Y33W
12+ 799.4
11+ 872.1
13+ 738.1
14+ 685.4
10+ 959.3
9+ 1065.4
8+ 1198.7
7+ 1369.9
6+
9581.6
L
S14D-Y33W-H49K
12+ 798.6
11+ 871.2
13+ 737.4
8+ 1197.6
7+ 1368.7
10+ 958.3
9+ 1064.6
14+ 684.7
9572.2

Fig. S3. ESI-MS spectra of recombinant bovine and human IF_1 proteins. Each row shows the raw spectra and the transformations of these data onto a molecular mass scale. (*A*) BovIF$_1$(1-84)-Y33W; (*B*) BovIF$_1$(1-84)-Y33W-H49K; (*C*) BovIF$_1$(1-62)-Y33W; (*D*) BovIF$_1$(1-62)-Y33W-H49K; (*E*) HumIF$_1$(1-81)-wild-type; (*F*) HumIF$_1$(1-81)-Y33W; (*G*) HumIF$_1$(1-81)-H49K; (*H*) HumIF$_1$(1-81)-Y33W-H49K; (*I*) HumIF$_1$(1-81)-S14D-Y33W; (*J*) HumIF$_1$(1-81)-S14D-Y33W-H49K; (*K*) HumIF$_1$(1-81)-S14E-Y33W; (*L*) HumIF$_1$(1-81)-S14E-Y33W-H49K.

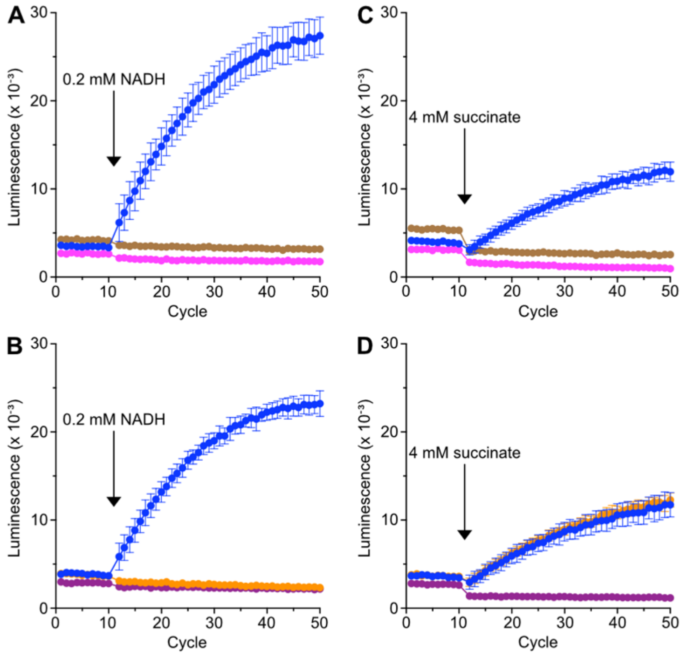

Fig. S4. Inhibition of ATP synthesis in bovine SMPs by respiratory inhibitors. ATP synthesis coupled to oxidation of (*A* and *B*), NADH (0.2 mM) or (*C* and *D*), succinate (4 mM). ATP synthesis in the presence of (●), DMSO vehicle only; (●), 1 µM oligomycin; (●), 0.5 µM FCCP; (●), 1 µM rotenone; (●), 1 µM antimycin A. Background luminescence levels were established for 10 measurement cycles before addition of substrate and generation of ATP. Measurements of luminescence were made in quadruplicate and average values ± SD are shown.

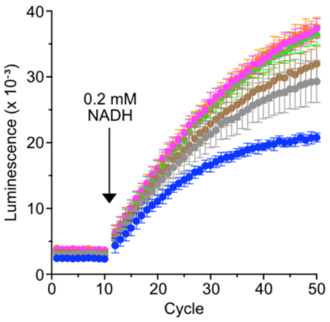

Fig. S5. Effect of the concentration of BovIF$_1$(1-84)-Y33W on the synthetic activity of ATP synthase. (●), no inhibitor; (●), 1.35 μM, (●), 6.75 μM; (●), 33.8 μM; (●), 67.5 μM; (●), 135 μM. At a concentration of 135 μM inhibitor protein, the molar ratio with respect to the ATP synthase is ca. 11000:1. ATP synthesis was initiated by the addition of NADH, and monitored by a luminescence continuous real-time assay with a luciferase-luciferin reagent. Background luminescence levels were established for 10 measurement cycles. N=4 wells, and data points show the average signal ± SD.

A

```
                 10         20         30         40         50         60         70         80
                        H1           H2
Bt   1 GSESGDNVRS SAGAVRDAGG AFGKREQAEE ERYFRARAKE QLAALKKHHE NEISHHAKEI ERLQKEIERH KQSIKKLKQS EDDD- 84
Hs   1 GSDQSENVDR GAGSIREAGG AFGKREQAEE ERYFRAQSRE QLAALKKHHE EEIVHHKKEI ERLQKEIERH KQKIKMLKHD D---- 81
Pa   1 GSDQSENVDR GAGSIREAGG AFGKREQAEE ERYFRAQSRE QLAALKKHHE EEIVHHKKEI ERLQKEIERH KQKIKMLKHD D---- 81
Cap  1 GSDQSENVDR GAGAIREAGG AFGKREQAEE ERYFRTQSRE QLAALKKHHE EEIVHHKKEI ERLQKEIERH KQKIKTLKHD D---- 81
Ss   1 SSDTPEGVRS GAGAVRDAGG AFGKKEQADE ERYFRARARE QLAALKKHHE NEISHHVKEI ERLQKEIERH KQSIKKLKND DDD-- 83
Ec   1 SSDRSKDHDS PAGSIREAGG AFGKREQAEE ERYFRKRTRE QLAALKKHHE DEISHHVKEI EHLQKEIERH KQRIKTLKDH DDD-- 83
La   1 SSDQPHDLDR GAGAIREAGG AFAKREAAEE ERYFRAQAKE QLAALKKYHE EEITHTKKEI ERLQNEIERH KQKIKKLKHD D---- 81
Cf   1 SSDRPDSVRP SEGAVRDAGG AFGKREQAEE ERYFRARTRE QLAALKKHHE NEISHHHQEI EHLQKEIERH KQAIKKLKQH DDD-- 83
Clf  1 GSERSEDP-G SAGSIREAGG AFGKREQAEE ERYFRARTRE QLAALKKHHE DEISHHIKEI ERLQKEIERH KKKIKNLKSD DD--- 81
Fc   1 SSERSEDS-- RAGSIREAGG AFGKREQAEE ERYFRARTRE QLAALKKHHE DEISHHIKEI ERLQKEIERH KKKIKHLKRD EEEDD 83
Mpf  1 SSERPDS--- SAGSIREAGG AFGKREQAEE ERYFRARTKE QLAALKKHHE EEITHHLKEI ERLQKEIERH KKKIKHLKDD DD--- 79
Cp   1 GSDKD--HDG SSGSIREAGG AFGKREQAEE ERFFRQKTRE QLEALKKHHE DEIYHHKKEI ERMEKEIERH KQKIKQLKHD ----- 78
Mm1  1 GSDQSEDVDR SAGAIREAGG AFGKRERAEE ERYFREKTRE QLAILKKHHE DEISHHKKEI ERLQKEIERH KQKIKHLHND D---- 81
It   1 GSDQSEDGGR SAGAIREAGG AFGKRERAEE ERYFREKTRE QLAILKKHHE DEISHHKKEI ERLQKEIERH KQKIKHLHND D---- 81
Rn   1 GSDSSESMDS GAGSIREAGG AFGKREKAEE DRYFREKTRE QLAALKKHHE DEIDHHSKEI ERLQKQIERH KKKIKYLKNS EH--- 82
Mm2  1 VSDSSDSMDT GAGSIREAGG AFGKREKAEE DRYFREKTKE QLAALRKHHE DEIDHHSKEI ERLQKQIERH KKKIQQLKNN H---- 81
Ml   1 GCGRSARFST DSGAIKEAGG SFARREKAEE ERYFLERTKE QLAALKKHHE EEINHHKKEI ERLQKEIERH KQNIKKLKDH GDD-- 83
Mm3  1 GSDRSN---- SAGAVREAGG AFGKREQAEE ERYFRARTKE QLAALKKHHE DEITHYTKEI ERLQKEIERH KHTIKKLKDT AGD-- 78
Tt   1 GSESADNLGS SAGAVRDAGG AFGKKEQAEE ERYFRARAKE QLAALKKHHE NEISHHMKEI EHLQKEIERH KQSIKKLKRD DD--- 82
Bas  1 GSESADNLGS SAGAVRDAGG AFGKKEQAEE ERYFRARAKE QLAALKKHHE NEISHHVKEI EHLQKEIERH KQSIKKLKRD DD--- 82
          .          *::::*** :*.::* *:* :*:*   :::* **  *:*:** :** *   :** *:::::**** *: *: *:
```

B

```
               10                20         30         40         50         60         70         80
Hs  1 GSDQSENVDR GAG-------SIREAGG AFGKREQAEE ERYFRAQSRE QLAALKKHHE EEIVHHKKEI ERLQKEIERH KQKIKMLKHD D 81
Cm  1 GSDQLGELGS GAGKGGGGGGSIREAGG AFGKRQAAHE ERYFREKEAE QLASLRKHHE EEIDHHKKEI ERLQKEIERH KYKIKKLKND D 88
Zv  1 GADQLGGLGQ GAGKGGGGGGSIRDAGG AFGKREAALE ERYFREKGNE QLAELKKHHE DEIYHQKKEI ERLQKEIERH KYKLKKLTGD D 88
Pb  1 GSDQLGELGK GAGKGGGGGGTIREAGG AFGKREAAAE ERYFKEKERE QLANLKRYHE EEISHHKKEI ERLQKEIERH KSKIKKLDDD - 87
Gg  1 GADQLGELGK GAGKGGGGGGSIREAGG AFGKKQAAEE ERYFREKERE QLAALRRHHE EEIDHHKKEI ERLQKEIERH KHKIKKLKDD D 88
Cs  1 GADQLGELGK GAGKGGGGGGTIREAGG AFGKKQAAEE ERYFREKERE QLAALRRHHE EEIDHHKKEI ERLQKEIERH KHKIKKLKDD D 88
Ap  1 GADQLGELGK GAGKGGGGGGAIREAGG AFGKKQAAEE ERYFREKERE QLAALRKHHE EEIDHHKKEI ERLQKEIERH KYKIKKLKDD D 88
Xt  1 SSDQLGELGK GAGKGGGGGGSVREAGG AFGKRQAAEE ERYFRQKEQE QIASLRKHHE EEIRHHKGEI ERLQKEIERH KSKIKKLNDD - 87
Rt  1 SSDQLGELGK GAGKGGGGGGSVREAGG AFGKRQAAEE ERYFKQKERE QIDSLKKHYE EEIHHHKDEI ERLQKEIERH KGKIKKLKHD D 88
Gs  1 DSSQLGELGK GAGKGGGGGGTVREAGG AFGKREAAEE ERYFKQKERE QLSALQKHHR EEITHYEKEI ERLHKQIERH KCKIKELRDD - 86
Dr  1 SSDQLGELGT GAGKGGGGGGSVRAAGG SFGRREAAEE ERYFRQKERE QLAALKNHHE EEIDHHKKEI ERLQREIDRH KGKIRKLKHD D 88
El  1 ASDQLGELGK GAGKGGGGGGTVREAGG AFGKREAAEE EYYFRQKEKE QLAALRKHHE EEIEHSKKEI ERLQREIDRH KGKIKKLKHD D 87
Cv  1 SSDQLGELGK GAGRGGGGGGYVRSAGG AFGKREAAEE ERYFRERERE QLEALRKHHA EEIEHHKKEI ERLQKEIDRH KGKIRKLSHD D 88
        :.*   :.  ***        :* *** :**::: * * * **: :   * *:  *:.::  :** * : ** ***:::*:** * *:: *  *
```

Fig. S6. Comparison of sequences of IF_1 proteins from various vertebrates. The N-terminal residues of only the mature bovine and human proteins have been identified experimentally. The mitochondrial import precursors of the inhibitors from other species are assumed to be processed at the same site. Residue Ser-14 in the human protein has been proposed to be phosphorylated reversibly. This residue and conserved serine residues in the same position in other species are shown in red. The symbols *, : and . denote identical, strongly conserved and weakly conserved residues respectively. (*A*) Sequences of mammalian IF_1 molecules. The grey bars H1 and H2 denote the positions of α-helices in the structure of the bovine protein. Bt, *Bos taurus* (cattle, sp|P01096); Hs, *Homo sapiens* (human, sp|Q9UII2); Pa, *Pongo abelii* (Sumatran orangutan, sp|Q5RFJ9); Cap, *Colobus angolensis palliatus* (Angolan colobus, tr|A0A2K5KBI5); Ss, *Sus scrofa* (pig, sp|Q29307); Ec, *Equus caballus* (horse, tr|F6ZXT0); La, *Loxodonta africana* (African bush elephant, tr|G3SWQ8); Cf, *Camelus ferus* (wild Bactrian camel, tr|S9XNE5); Clf, *Canis lupus familiaris* (domestic dog,

tr|E2QYN4); Fc, *Felis catus* (cat, tr|M3WIS8); Mpf, *Mustela putorius furo* (ferret, tr|M3YVR5); Cp, *Cavia porcellus* (guinea pig, tr|A0A286Y431); Mm1, *Marmota monax* (groundhog, tr|A0A5E4AD98); It, *Ictidomys tridecemlineatus* (thirteen-lined ground squirrel, tr|I3N8E6); Rn, *Rattus norvegicus* (brown rat, sp|Q03344); Mm2, *Mus musculus* (mouse, sp|O35143); Ml, *Myotis lucifugus* (little brown bat, tr|G1NSN7); Mm3, *Molossus molossus* (Pallas's mastiff bat, tr|A0A7J8F6B1); Tt, *Tursiops truncates* (common bottlenose dolphin, tr|A0A2U3V0R3); Bas, *Balaenoptera acutorostrata scammoni* (minke whale, tr|A0A383Z6R7). (*B*) The sequence of human IF_1 compared with examples of orthologs from reptiles, birds, amphibians and fish. Hs, *Homo sapiens* (human, sp|Q9UII2); Cm, *Chelonia mydas* (green sea turtle, ncbi|XP_037737775); Zv, *Zootoca vivipara* (European common lizard, ncbi|XP_034976781); Pb, *Python bivittatus* (Burmese python, ncbi|XP_007434913); Gg, *Gallus gallus* (chicken, ncbi|XP_015153068); Cs, *Callipepla squamata* (scaled quail, ncbi|OXB61861); Ap, *Anas platyrhynchos* (mallard duck, ncbi|XP_027299537); Xt, *Xenopus tropicalis* (western clawed frog, sp|F7BK26); Rt, *Rana temporaria* (European common frog, ncbi|XP_040193223); Gs, *Geotrypetes seraphini* (Gaboon caecilian, ncbi|XP_033813241); Dr, *Danio rerio* (zebrafish, sp|A3KNL5); El, *Esox lucius* (northern pike, tr|C1BWJ3); Cv, *Cyprinodon variegatus* (sheepshead minnow, tr|A0A3Q2D9N0). Sequences were taken from the Swiss-Prot (sp), TrEMBL (tr) and NCBI (ncbi) databases. The alignments were made with Clustal Omega (1.2.4). In (*B*) the alignment was adjusted manually post Clustal Omega, placing the gap in the human sequence with the run of Gly residues found in the other proteins.

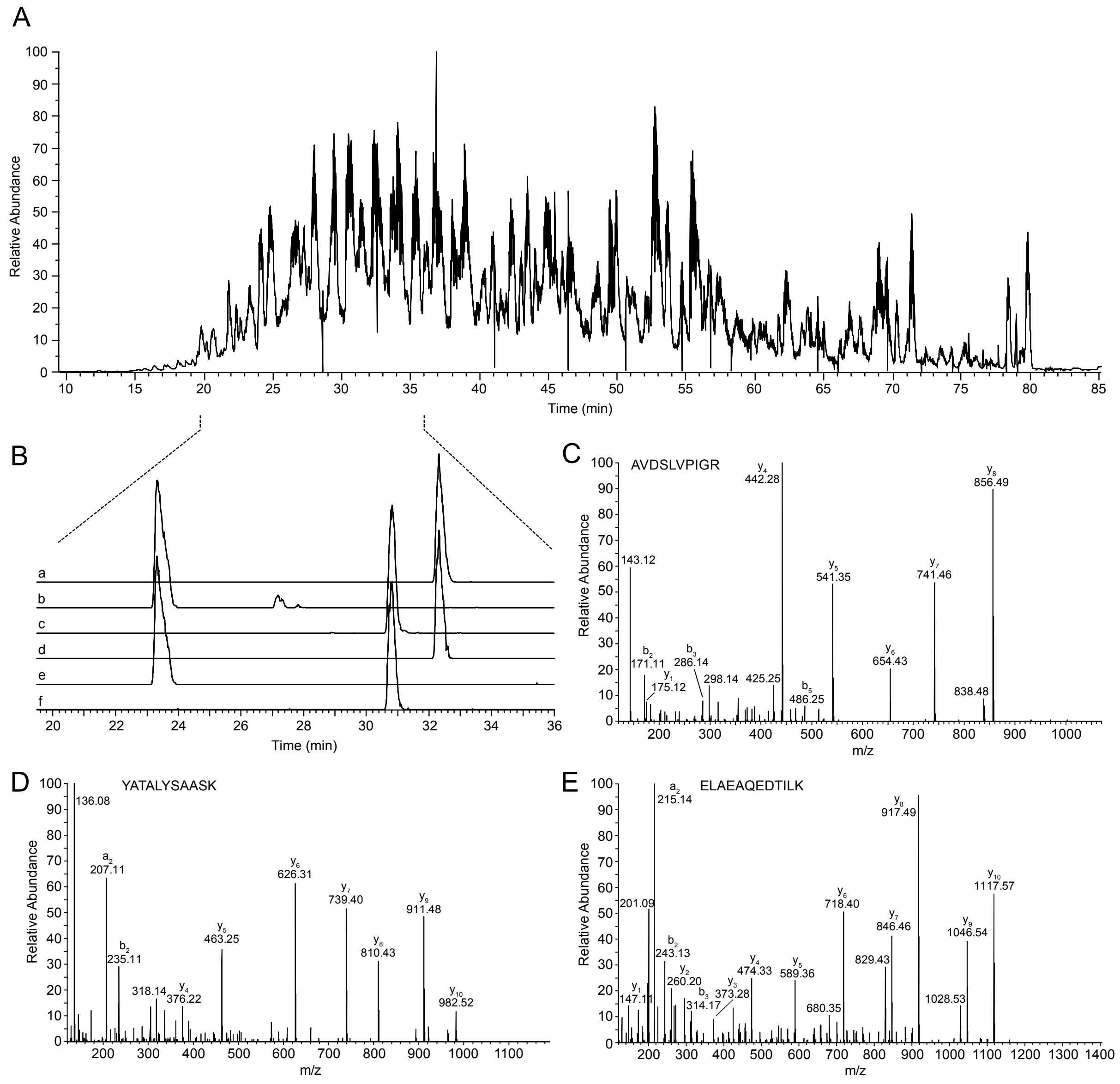

Fig. S7. The abundance of ATP synthase in bovine submitochondrial particles (SMPs) determined by quantitative mass spectrometry. Examples of: (*A*) LC-MSMS total ion current from an analysis of a tryptic digest of SMPs, spiked with heavy labelled subunit specific peptides (see Table S5). (*B*) Extracted ion chromatograms showing co-eluting peaks for the subunit specific labelled and endogenous peptides (charge state 2^+) of subunit-α (a and d), OSCP (b and e) and subunit-e (c and f). (*C*-*E*) Tandem-MSMS spectra providing identification of the endogenous peptides.

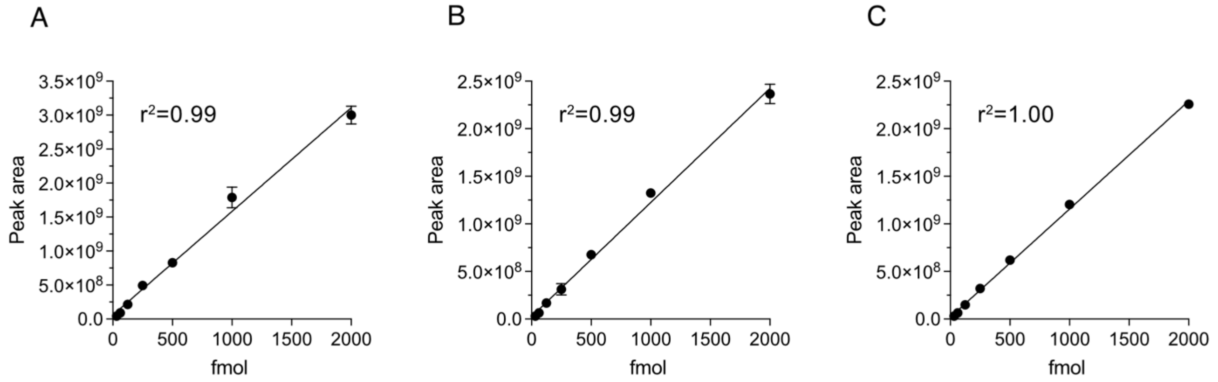

Fig. S8. Peak areas versus standard peptide concentration. A serial dilution of a mixture of three heavy labelled reference tryptic peptides (see Table S5) for ATP synthase were analyzed using an Orbitrap QE mass spectrometer (ThermoFisher). (*A*) α-subunit, (*B*) OSCP and (*C*) and subunit-e with charge state 2^+ were obtained from the extracted ion chromatograms of the parent ions with Xcalibur (ThermoFisher) and an m/z tolerance of 5 ppm. N=2, ± SD.

Table S1. Residues involved in binding bovine IF_1 to the β-, α- and γ-subunits of bovine F_1-ATPase and the identities of the equivalent amino acids in the human orthologs

IF_1		β-subunit		α-subunit		γ-subunit	
bovine	human	bovine	human	bovine	human	bovine	human
S11	G11					N15	N15
A12	A12			E353	E353		
G13	G13	D386	D386				
V15	I15	D386	D386				
D17	E17	D386	D386				
F22	F22	D386<br>I390<br>L391	D386<br>I390<br>L391			I16	I16
R25	R25					E241	E241
E30	E30	R408	R408				
E31	E31	R408	R408				
Y33	Y33	K401<br>M393<br>D394	K401<br>M393<br>D394				
F34	F34	V404<br>S405<br>R408<br>E454	V404<br>S405<br>R408<br>E454				
R35	R35			E399	E399		
Q41	Q41	D450	D450				
L42	L42	P453<br>L473<br>A474<br>H477	P453<br>L473<br>A474<br>H477				
L45	L45	A470<br>D471<br>A474	A470<br>D471<br>A474				

The bovine data are taken from Bason *et al* (2014) (1).

Table S2. Content of ATP synthase in bovine SMPs

Experiment[1]	ATP synthase		Subunit ratio
	nmol/mg SMP ± SD	mg/mg SMP ± SD	α : OSCP : e
A	0.49 ± 0.09	0.28 ± 0.05	3.0 : 0.9 : 1.4
B	0.47 ± 0.05	0.27 ± 0.03	3.0 : 0.9 : 1.1
Average	0.48	0.28	3.0 : 0.9 : 1.2

[1] For experiment A, two samples of the same preparation of extracted SMPs were digested separately with trypsin and each analyzed twice by MS. The ratio is an average of the four calculated values. For experiment B, a single digest was performed, analyzed twice by MS, with the ratio an average of the two calculated values. Data file S27.

Table S3. Molar ratios of IF_1 and ATPase in various animals and tissues estimated by quantitative Western blotting

Molar ratio	Sample	Method	Reference
2.5:1	Goat heart mitochondria	Western blotting	(2)
4-6:1	Human heart and brain tissue	Western blotting	(3)
0.5-1:1	Human liver and kidney tissue	Western blotting	(3)
4-9:1	Mouse kidney, brain and colon tissue	Western blotting	(3)
0-1:1	Mouse heart and liver tissue	Western blotting	(3)

Table S4. Molecular masses of bovine and human IF_1 and mutant forms determined by electrospray mass spectrometry

Protein	Mass (Da)		Mass Difference (Da)
	Expected	Observed	
$BovIF_1$(1-84)-Y33W	9604.5	9603.6	-0.9
$BovIF_1$(1-84)-Y33W-H49K	9595.3	9594.5	-0.8
$BovIF_1$(1-62)-Y33W	6926.5	6926.2	-0.3
$BovIF_1$(1-62)-Y33W-H49K	6917.6	6917.1	-0.5
$HumIF_1$(1-81)-wild-type	9516.6	9516.3	-0.3
$HumIF_1$(1-81)-Y33W	9539.6	9539.5	-0.1
$HumIF_1$(1-81)-H49K	9507.6	9506.9	-0.7
$HumIF_1$(1-81)-Y33W-H49K	9530.6	9530.1	-0.5
$HumIF_1$(1-81)-S14D-Y33W	9567.6	9566.9	-0.7
$HumIF_1$(1-81)-S14D-Y33W-H49K	9558.6	9558.5	-0.1
$HumIF_1$(1-81)-S14E-Y33W	9581.6	9581.6	0
$HumIF_1$(1-81)-S14E-Y33W-H49K	9572.7	9572.2	-0.5

Table S5. Reference tryptic peptides for subunits of bovine ATP synthase

Subunit	Sequence (residue numbers)	Mass	
		Unlabelled	Heavy Labelled
α	AVDSLVPIGR (152-161)[1]	1025.5869	1032.6041
OSCP	YATALYSAASK (18-28)[1]	1144.5764	1151.5936
e	ELAEAQEDTILK (59-70)[1]	1358.6929	1365.7101

[1]With ^{13}C and ^{15}N at leucine residues 156, 22 and 69, respectively.

Table S6. Determination of subunit abundance and ratio data

Sequence	Labelled peptide		Endogenous peptide			Subunit ratio	F_1F_o nmol/mg
	MH^{2+}	Area	MH^{2+}	Area	fmol		
AVDSLVPIGR	517.3093	2550550439	513.8007	21750821234	1731.2	3.00	0.451
YATALYSAASK	576.8041	5329160543	573.2955	13880112315	528.7	0.92	0.413
ELAEAQEDTILK	683.8623	2921375675	680.3537	10941705354	760.3	1.32	0.594

The Table contains a representative example of data obtained from an LC-MSMS analysis of a tryptic digest of SMPs (1.28 μg), spiked with heavy labelled subunit specific peptides (203 fmol; see Table S5). Peak area ratios were obtained from the extracted ion chromatograms of the co-eluting peaks for the subunit specific peptides (charge state 2^+) and the amount of endogenous peptide was calculated from the known quantity of labelled peptide. The subunit ratio was calculated based on the presence of three α-subunits in each enzyme complex.

References

1. Bason, J. V., Montgomery, M. G., Leslie, A. G. W. and Walker, J. E. (2014) Pathway of binding of the intrinsically disordered mitochondrial inhibitor protein to F_1-ATPase. *Proc. Natl. Acad. Sci. U S A* **111**, 11305-11310

2. Di Pancrazio, F., Mavelli, I., Isola, M., Losano, G., Pagliaro, P., Harris, D. A. and Lippe, G. (2004) In vitro and in vivo studies of F_oF_1 ATP synthase regulation by inhibitor protein IF_1 in goat heart. *Biochim. Biophys. Acta* **1659**, 52-62

3. Esparza-Moltó, P. B., Nuevo-Tapioles, C., Chamorro, M., Nájera, L., Torresano, L., Santacatterina, F. and Cuezva, J. M. (2019) Tissue-specific expression and post-transcriptional regulation of the ATPase inhibitory factor 1 (IF1) in human and mouse tissues. *FASEB J.* **33**, 1836-1851
